# The use of mono- and combination drug therapy in men and women with lower urinary tract symptoms (LUTS) in the UK: a retrospective observational study

**DOI:** 10.1186/s12894-021-00881-w

**Published:** 2021-09-02

**Authors:** Mahmood Ali, Margarita Landeira, Patrick J. O. Covernton, Nurul Choudhury, Ashley Jaggi, Francis Fatoye, Rob van Maanen

**Affiliations:** 1 Manchester Metropolitan University, Manchester, UK; 2 Present Address: Astellas Pharma Europe Ltd, Addlestone, UK; 3 Astellas Global Development, Leiden, The Netherlands

**Keywords:** Benign prostatic obstruction (BPO), Overactive bladder (OAB), Lower urinary tract symptoms (LUTS), Persistence, Stress urinary incontinence (SUI)

## Abstract

**Background:**

Combination drug therapy for lower urinary tract symptoms (LUTS) is beneficial to selected patients and recommended by guidelines. Patterns of real-world LUTS drug use, especially combination drug therapy, have not been studied extensively. Moreover, further understanding of the recent landscape is required following the introduction of the beta-3-adrenoceptor agonist mirabegron in the UK in 2013 for overactive bladder (OAB). The objective was to describe mono- and combination drug therapy use for LUTS in patients in UK clinical practice.

**Methods:**

This was a retrospective, descriptive, observational database study using UK Clinical Practice Research Datalink GOLD and linked databases. Men and women ≥ 18 years with a first prescription for any LUTS drug from 2014 to 2016 with ≥ 12 months continuous enrollment pre- and post-index date were included. Primary endpoints were mono- or combination drug therapy use for LUTS in male and female cohorts. Secondary endpoints were description of treatment prescribed, treatment persistence and patient demographics. Data were analyzed descriptively. Sub-cohorts were defined by drugs prescribed at index date.

**Results:**

79,472 patients (61.3% male) were included, based on index treatments. Of all men, 82.5% received any benign prostatic obstruction (BPO) drug, 25.4% any OAB drug, and 7.9% any BPO drug plus any OAB drug. As either mono- or combination drug therapy, 77.1% received an alpha-blocker, 18.9% a 5-alpha reductase inhibitor, 23.9% an antimuscarinic agent, and 2.1% mirabegron. Of all women, 94.5% received any OAB drug, 6.0% duloxetine, and 0.5% any OAB drug plus duloxetine. As either mono- or combination drug therapy, 87.7% received an antimuscarinic, and 9.7% mirabegron. In men or women receiving OAB treatment, approximately 2.5% received combination drug therapy with an antimuscarinic agent and mirabegron. For OAB drug monotherapies, mirabegron had the highest persistence in both male and female cohorts.

**Conclusions:**

This study provides a better understanding of the recent landscape of LUTS drug use in UK clinical practice. It highlights potential undertreatment of storage symptoms in men with LUTS and the low use of combination OAB treatments.

**Supplementary Information:**

The online version contains supplementary material available at 10.1186/s12894-021-00881-w.

## Background

Lower urinary tract symptoms (LUTS) is an overarching term for symptoms in men and women, comprising storage, voiding and post-voiding components [1, 2]. In both men and women, storage LUTS are commonly attributed to overactive bladder (OAB) syndrome [1–3], which is defined as urinary urgency, usually with increased daytime frequency and/or nocturia, with/without urinary incontinence, and with no urinary tract infection or other detectable disease [1, 2, 4]. Stress urinary incontinence (SUI) is another common cause of LUTS, especially in women, and involves involuntary urine leakage associated with physical activity (e.g., coughing, sneezing), often as a consequence of childbirth [1, 3, 5]. Voiding LUTS in men are commonly attributed to benign prostatic obstruction (BPO: bladder outlet obstruction [BOO] due to benign prostatic enlargement) [1, 6].

Although conservative treatment, including lifestyle intervention and behavioral therapies (such as bladder training), remains the foundation of LUTS management, several pharmacological treatments are available [6–8]. If conservative treatment fails, pharmacological therapy for OAB/urgency urinary incontinence (UUI) includes antimuscarinic agents or the beta-3 agonist, mirabegron [7]. Combination of an antimuscarinic agent plus mirabegron has also been shown to be effective [9] and is recommended as an option if patients respond inadequately to monotherapy [7, 8]. In patients with SUI where surgery is not indicated, duloxetine is the only recommended pharmacotherapy [7]; it is unknown to what extent duloxetine is used in combination with OAB drugs. Women with LUTS can experience symptoms of both OAB and SUI [10], but there are no recommendations regarding combination therapy with OAB/UUI drugs for such patients.

In men with LUTS suggestive of BPO, the main treatment options include alpha-blockers for rapid symptomatic relief [11, 12] and 5-alpha reductase inhibitors (5-ARIs) to delay progression of BPO and help manage symptoms over the long term in men at risk of disease progression [6, 12]. Alpha-blockers are usually used as first-line treatment [6], but the European Association of Urology (EAU) guidelines recommend combined alpha-blocker and 5-ARI treatment in men with moderate-to-severe LUTS and an increased risk of disease progression [6]. However, many men with LUTS (approximately 50%) experience mixed symptoms suggestive of both OAB and BPO [13]. Only one-third of these men with mixed symptoms will achieve adequate symptom control with an alpha-blocker alone, with the remainder requiring additional pharmacotherapy to manage residual storage LUTS [14]. Clinical trials in men on alpha-blocker monotherapy who still have bothersome storage LUTS have shown that adding an OAB drug can significantly reduce storage symptoms and improve quality of life [15–18]. The addition of an antimuscarinic agent or beta-3-agonist if storage symptoms are not relieved by alpha-blocker monotherapy is a recommended treatment strategy in clinical guidelines [6].

Patterns of real-world LUTS drug use, especially combination drug therapy, have not been studied extensively in the UK. Our study investigated the recent landscape of pharmacotherapy for men and women with LUTS in UK clinical practice, including the types and extent of combination therapies used, and persistence with treatment.

## Methods

### Study design

This was a retrospective, descriptive, observational database study of LUTS treatment in the UK primary care setting. Data were extracted from the UK Clinical Practice Research Datalink (CPRD) GOLD, a national longitudinal primary care database, which contains anonymized electronic health records of over 15.6 million patients (September 2018 version) [19]. Only de-identified data were obtained, and patients could opt out if they did not wish to have their data used for research purposes. CPRD GOLD was linked to the Hospital Episode Statistic database in England for the exploration of resource use, and the Index of Multiple Deprivation (IMD). The IMD provides an indication of patients’ socio-economic status measured at the GP surgery level. All methods were carried out in accordance with relevant guidelines and regulations.

### Population

The study included adults (≥ 18 years of age) identified with LUTS (evidenced by prescription of drugs used to treat LUTS) between 1 January 2014 and 31 December 2016, with ≥ 12 months continuous enrollment pre- and post-index date (index date was the date on which patients were prescribed a new index pharmacotherapy [one pharmacotherapy or combination of two or more pharmacotherapies] for the first time between 1 January 2014 and 31 December 2016). Patients prescribed the same index drug in the 12-month pre-index period were excluded. Male and female cohorts were considered separately. Sub-cohorts were defined by the drugs received at index date (Additional file 1: Table S1), and combination therapy was categorized by index drug with patients placed into one sub-cohort only. If > 1 drug was prescribed at index date and this indicated assignment to different sub-cohorts, the order of preference was as follows: LUTS (including OAB) had priority over BPO in the male cohort and LUTS (including OAB) over SUI in the female cohort. Patients were classified as being on combination therapy if the additional drug(s) was prescribed within the prescription duration of the first drug (prescription duration was calculated by total tablets prescribed divided by total daily dose) and the index date was the first prescription of the most recently prescribed drug.

The LUTS (including OAB) cohort were patients receiving an OAB drug (antimuscarinic agent and/or mirabegron), with or without a BPO drug (alpha-blocker and/or 5-ARI) in the male cohort and with or without a SUI drug (duloxetine) in the female cohort. BPO patients were those receiving an alpha-blocker and/or 5-ARI without an OAB drug. SUI patients were those receiving duloxetine without an OAB drug (Additional file 1: Table S1).

### Endpoints

Primary endpoints were use of mono- or combination drug therapy for LUTS in a) male or b) female cohorts. Secondary endpoints were socio-demographic and clinical characteristics, and description of monotherapy/combination drugs. Exploratory variables included treatment persistence and presence of a LUTS diagnostic Read code in the pre-index period.

### Data analyses

Socio-demographic characteristics were recorded by age, sex and IMD; clinical characteristics were recorded by comorbidity (number of chronic diseases from the Quality and Outcomes Framework [QOF]), polypharmacy (number of distinct British National Formulary headers from CPRD GOLD), and antimuscarinic treatment experience (≥ 1 antimuscarinic agent prescription, other than index treatment, in CPRD GOLD), within the 12-month pre-index period.

### Statistical analyses

Data were analyzed descriptively for the overall population and by cohort and sub-cohort. Analyses were conducted using SAS Studio version 3.5.

Persistence was analyzed by median time (from index date) to first discontinuation (TTD, during the 12-month post-index period), and persistence rate at 12 months, calculated using the Kaplan–Meier method. Discontinuation was defined as exceeding the maximum allowable gap duration (MAGD) between prescriptions. For the base-case, the MAGD was defined as 1.5 times the estimated duration of the most recent prescription. Combination treatment was classed as discontinued upon discontinuation of any one of the component drugs. Data were classed as Not Observable when the number of patients still at risk was below the 20% of the initial sample threshold required to allow for persistence to be calculated or, the median was not reached.

### Sensitivity analyses

Sensitivity analyses (SA1-7) varied the combination drug therapy definition, MAGD definition and presence of LUTS diagnostic code. The combination drug therapy definition was changed to: a second drug of interest is prescribed within 1.5 times the estimated prescription interval of the first drug (SA1); two drugs of interest are prescribed on the same day (SA2); both drugs continue to be prescribed for ≥ 90 days from index date (SA3). MAGD definition was changed to: equivalent to (SA4) or double (SA5) the length of most recent prescription. To be considered a patient with LUTS, patients required a LUTS diagnosis code (Additional file 1: Tables S2a–c) registered any time before the index date (SA6). In the final sensitivity analysis, patients diagnosed with hypertension were excluded (SA7).

## Results

### Patients

**Fig. 1:**
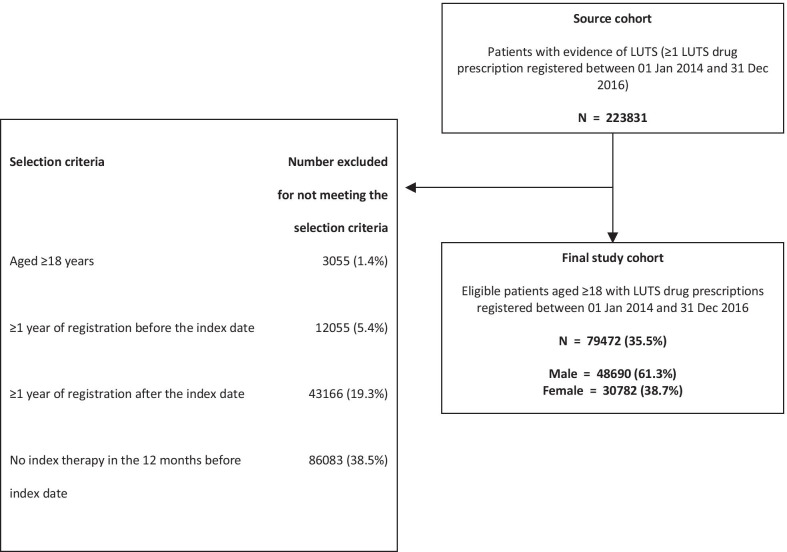
Selection criteria and study cohorts

Of 223,831 patients with one or more LUTS drug prescription between 1 January 2014 and 31 December 2016, 79,472 were included in the study (61.3% [*n* = 48,690] in the male and 38.7% [*n* = 30,782] in the female cohort) (Fig. 1) (Additional file 1: Table S3a and b).

At index date, the mean age was 65.5 years. In the 12-month pre-index period, patients had a mean of 0.2 new diagnoses of QOF chronic diseases and were prescribed a mean of 9.6 drugs; 5.1% of patients had received an antimuscarinic agent (Tables 1, 2). Approximately one in five patients (16.3%) had a LUTS diagnostic code, the notable exceptions being for doxazosin and duloxetine monotherapy (3% each).

**Table 1:** Population demographic and clinical characteristics for male sub-cohorts

		LUTS (including OAB) N = 12,383	BPO only N = 36,307
*Age at index date*	n	12,383	36,307
	Mean (SD)	65.54 (15.73)	67.98 (12.47)
	18–24	192 (1.6%)	119 (0.3%)
	25–34	434 (3.5%)	356 (1.0%)
	35–44	736 (5.9%)	997 (2.7%)
	45–54	1438 (11.6%)	3586 (9.9%)
	55–64	2188 (17.7%)	7746 (21.3%)
	65–74	3379 (27.3%)	11,905 (32.8%)
	≥ 75	4016 (32.4%)	11,598 (31.9%)
*Index of multiple deprivation at index prescription (GP surgery level)*	n	5112	16,534
	1 = least deprived	633 (12.4%)	2770 (16.8%)
	2	1124 (22.0%)	3654 (22.1%)
	3	968 (18.9%)	3175 (19.2%)
	4	963 (18.8%)	2926 (17.7%)
	5 = most deprived	1424 (27.9%)	4009 (24.2%)
*New comorbidities*	n	12,383	36,307
Count of newly diagnosed chronic diseases from the QOF within the 12-month pre-index period	Mean (SD)	0.21 (0.50)	0.22 (0.51)
	0	10,228 (82.6%)	29,480 (81.2%)
	1	1790 (14.5%)	5700 (15.7%)
	2	313 (2.5%)	978 (2.7%)
	3+	52 (0.4%)	149 (0.4%)
*Polypharmacy*	n	12,383	36,307
Number of distinct BNF headers within the 12-month pre-index period	Mean (SD)	9.81 (7.27)	8.52 (6.45)
	0	576 (4.7%)	1757 (4.8%)
	[1; 3]	1931 (15.6%)	6668 (18.4%)
	[4; 7]	2936 (23.7%)	10,342 (28.5%)
	[8; 19]	5707 (46.1%)	15,220 (41.9%)
	20+	1233 (10.0%)	2320 (6.4%)
*Antimuscarinic treatment experience within the 12-month pre-index period*	n	12,383	36,307
	Yes	908 (7.3%)	397 (1.1%)
	No	11,475 (92.7%)	35,910 (98.9%)

Male LUTS (including OAB) sub-cohort patients were those receiving an OAB drug (antimuscarinic and/or mirabegron) with or without a BPO drug (either an alpha-blocker and/or 5-ARI). Male BPO sub-cohort patients were those receiving an alpha-blocker and/or 5-ARI without an OAB drug. Percentages may not total exactly 100 due to rounding

*BNF*: British National Formulary; *BPO*: benign prostatic obstruction; *GP*: general practitioner; *LUTS*: lower urinary tract symptoms; *OAB*: overactive bladder; *QOF*: Quality and Outcomes Framework; *SD*: standard deviation

**Table 2:** Population demographic and clinical characteristics for female sub-cohorts

		LUTS (including OAB) N = 29,094	SUI N = 1688
*Age at index date*	n	29,094	1688
	Mean (SD)	62.92 (16.77)	54.24 (16.29)
	18–24	604 (2.1%)	32 (1.9%)
	25–34	1121 (3.9%)	159 (9.4%)
	35–44	2536 (8.7%)	315 (18.7%)
	45–54	4835 (16.6%)	399 (23.6%)
	55–64	5264 (18.1%)	315 (18.7%)
	65–74	6446 (22.2%)	248 (14.7%)
	≥ 75	8288 (28.5%)	220 (13.0%)
*Index of multiple deprivation at index prescription (GP surgery level)*	n	11,999	665
	1 = least deprived	1534 (12.8%)	86 (12.9%)
	2	2418 (20.2%)	148 (22.3%)
	3	2292 (19.1%)	137 (20.6%)
	4	2285 (19.0%)	105 (15.8%)
	5 = most deprived	3470 (28.9%)	189 (28.4%)
*New comorbidities*	n	29,094	1688
Count of newly diagnosed chronic diseases from the QOF within the 12-month pre-index period	Mean (SD)	0.20 (0.48)	0.23 (0.53)
	0	24,327 (83.6%)	1374 (81.4%)
	1	3950 (13.6%)	248 (14.7%)
	2	712 (2.4%)	56 (3.3%)
	3+	105 (0.4%)	10 (0.6%)
*Polypharmacy*	n	29,094	1688
Number of distinct BNF headers within the 12-month pre-index period	Mean (SD)	10.77 (7.49)	11.55 (7.42)
	0	681 (2.3%)	29 (1.7%)
	[1; 3]	3766 (12.9%)	165 (9.8%)
	[4; 7]	6944 (23.9%)	384 (22.7%)
	[8; 19]	14,178 (48.7%)	873 (51.7%)
	20+	3525 (12.1%)	237 (14.0%)
*Antimuscarinic treatment experience within the 12-month pre-index period*	n	29,094	1688
	Yes	2681 (9.2%)	56 (3.3%)
	No	26,413 (90.8%)	1632 (96.7%)

Female LUTS (including OAB) sub-cohort patients were those receiving an OAB drug (antimuscarinic and/or mirabegron) with or without a SUI drug (duloxetine). Female SUI sub-cohort patients were those receiving duloxetine without an OAB drug. Percentages may not total exactly 100 due to rounding

*BNF*: British National Formulary; *GP*: general practitioner; *LUTS*: lower urinary tract symptoms; *OAB*: overactive bladder; *QOF*: Quality and Outcomes Framework; *SD*: standard deviation; *SUI*: stress urinary incontinence

### Extent of monotherapy and combination drug use

Of all men (*n* = 48,690), 82.5% received any BPO drug (alpha-blocker and/or 5-ARI), 74.6% received any (one or more) BPO drug without an OAB drug, 25.4% received any OAB drug (antimuscarinic agent and/or mirabegron) (17.5% received any [one or more] OAB drug without a BPO drug, and 7.9% received any BPO drug plus any OAB drug). As either mono- or combination therapy, 77.1% were receiving an alpha-blocker, 18.9% received a 5-ARI, 23.9% an antimuscarinic agent and 2.1% mirabegron. The most common drug received either as mono- or combination therapy was tamsulosin (61.2% of all men) and the most common combination was finasteride plus tamsulosin (8.6%, with or without additional drugs). Of all women (*n* = 30,782), 94.5% received any OAB drug, 6.0% received a SUI drug, and 0.5% received any OAB drug plus a SUI drug (Additional file 1: Table S3b). As either mono- or combination therapy, 87.7% were receiving an antimuscarinic agent and 9.7% mirabegron. The extent of drug class use is summarized in Tables 3, 4 and 5, and in Additional file 1: Tables S3b and 4.

**Table 3:** Extent of mono- and combination drug therapy use in the male LUTS (including OAB) sub-cohort

Monotherapy	N	% of monotherapy patients	% of all patients in male OAB sub-cohort	% of all male patients
Any OAB drug monotherapy	7946	100.0	64.2	16.3
Any antimuscarinic monotherapy	7443	93.7	60.1	15.3
Solifenacin	2759	34.7	22.3	5.7
Oxybutynin	2613	32.9	21.1	5.4
Tolterodine	1445	18.2	11.7	3.0
Mirabegron	503	6.3	4.1	1.0
Fesoterodine	285	3.6	2.3	0.6
Trospium	227	2.9	1.8	0.5
Flavoxate	49	0.6	0.4	0.1
Darifenacin	47	0.6	0.4	0.1
Propiverine	18	0.2	0.1	0.04

Combination drug therapy	N	% of combination drug therapy patients	% of all patients in male OAB sub-cohort	% of all male patients
Any combination drug therapy	4437	100.0	35.8	9.1
Any BPO drug + any OAB drug	3863	87.1	31.2	7.9
Any BPO drug + any antimuscarinic	3643	82.1	29.4	7.5
Any BPO drug + mirabegron	347	7.8	2.8	0.7
Any alpha-blocker + any OAB drug (± 5-ARI)	3538	79.7	28.6	7.3
Any alpha-blocker + any antimuscarinic (± mirabegron and/or 5-ARI)	3330	75.1	26.9	6.8
Any alpha-blocker + mirabegron (± antimuscarinic and/or 5-ARI)	326	7.3	2.6	0.7
Triple drug therapy (any alpha-blocker + any 5-ARI + any OAB drug)	958	21.6	7.7	2.0
Any OAB drug + any 5-ARI (± alpha-blocker)	1284	28.9	10.4	2.6
Any OAB drug + any 5-ARI (excl. alpha-blocker)	325	7.3	2.6	0.7
Mirabegron + any antimuscarinic (± BPO drug)	307	6.9	2.5	0.6
Any ≥ 2 OAB (± BPO drug)	964	21.7	7.8	2.0
Any ≥ 2 OAB (no BPO drug)	574	12.9	4.6	1.2
Any ≥ 2 antimuscarinics (± BPO drug and/or mirabegron)	666	15.0	5.4	1.4
Solifenacin + tamsulosin	902	20.3	7.3	1.9
Oxybutynin + tamsulosin	364	8.2	2.9	0.7
Tamsulosin + tolterodine	248	5.6	2.0	0.5
Finasteride + solifenacin + tamsulosin	233	5.3	1.9	0.5
Doxazosin + solifenacin	114	2.6	0.9	0.2
Solifenacin + tolterodine	114	2.6	0.9	0.2
Finasteride + oxybutynin + tamsulosin	111	2.5	0.9	0.2
Oxybutynin + solifenacin	102	2.3	0.8	0.2
Finasteride + solifenacin	96	2.2	0.8	0.2
Mirabegron + tamsulosin	96	2.2	0.8	0.2
Doxazosin + oxybutynin	95	2.1	0.8	0.2
Mirabegron + solifenacin	95	2.1	0.8	0.2
Fesoterodine + tamsulosin	83	1.9	0.7	0.2
Dutasteride + solifenacin + tamsulosin	79	1.8	0.6	0.2
Doxazosin + tolterodine	69	1.6	0.6	0.1
Other combinations	1636	36.9	13.2	3.4

Drug class	N	% of all patients in male OAB sub-cohort	% of all male patients
Any OAB drug	12,383	100	25.4
Any OAB drug (no BPO drug)	8520	68.8	17.5
Any antimuscarinic	11,653	94.1	23.9
Total mirabegron	1039	8.4	2.1
Any alpha-blocker	3538	28.6	7.3
Any 5-ARI	1284	10.4	2.6

*5-ARI*: 5-alpha reductase inhibitor; *BPO*: benign prostatic obstruction; *LUTS*: lower urinary tract symptoms; *OAB*: overactive bladder

**Table 4:** Extent of mono- and combination drug therapy use in the male BPO sub-cohort

Monotherapy	N	% of monotherapy patients	% of all patients in BPO sub-cohort	% of all male patients
Any BPO drug monotherapy	29,739	100.0	81.9	61.1
Any alpha-blocker monotherapy	27,462	92.3	75.6	56.4
Any 5-ARI monotherapy	2277	7.7	6.3	4.7
Tamsulosin	21,158	71.1	58.3	43.5
Doxazosin	5456	18.3	15.0	11.2
Finasteride	2131	7.2	5.9	4.4
Alfuzosin	665	2.2	1.8	1.4
Dutasteride	146	0.5	0.4	0.3
Prazosin	101	0.3	0.3	0.2
Terazosin	44	0.1	0.1	0.1
Indoramin	38	0.1	0.1	0.1

Combination drug therapy	N	% of combination drug therapy patients	% of all patients in BPO sub-cohort	% of all male patients
Any combination drug therapy	6568	100.0	18.1	13.5
Any alpha-blocker + any 5-ARI	5573	84.9	15.3	11.4
Finasteride + tamsulosin	3262	49.7	9.0	6.7
Dutasteride + tamsulosin	1189	18.1	3.3	2.4
Doxazosin + tamsulosin	614	9.3	1.7	1.3
Doxazosin + finasteride	289	4.4	0.8	0.6
Alfuzosin + finasteride	233	3.5	0.6	0.5
Alfuzosin + tamsulosin	208	3.2	0.6	0.4
Doxazosin + finasteride + tamsulosin	167	2.5	0.5	0.3
Dutasteride + finasteride + tamsulosin	125	1.9	0.3	0.3
Other combinations	481	7.3	1.3	1.0

Drug class	N	% of all patients in BPO sub-cohort	% of all male patients
Any BPO drug	36,307	100	74.6
Any alpha-blocker	33,984	93.6	69.8
Any 5-ARI	7896	21.7	16.2

*5-ARI*: 5-alpha reductase inhibitor; *BPO*: benign prostatic obstruction

**Table 5:** Extent of mono- and combination drug therapy use in the female LUTS (including OAB) sub-cohort

Monotherapy	N	% of monotherapy patients	% of all patients in female OAB sub-cohort	% of all female patients
Any OAB drug monotherapy	26,338	100.0	90.5	85.6
Any antimuscarinic monotherapy	24,263	92.1	83.4	78.8
Solifenacin	10,083	38.3	34.7	32.8
Oxybutynin	7852	29.8	27.0	25.5
Tolterodine	4020	15.3	13.8	13.1
Mirabegron	2075	7.9	7.1	6.7
Fesoterodine	1173	4.5	4.0	3.8
Trospium	754	2.9	2.6	2.4
Darifenacin	209	0.8	0.7	0.7
Flavoxate	96	0.4	0.3	0.3
Propiverine	76	0.3	0.3	0.2

Combination drug therapy	N	% of combination drug therapy patients	% of all patients in female OAB sub-cohort	% of all female patients
All combination drug therapy	2756	100.0	9.5	9.0
Any ≥ 2 OAB drugs	2621	95.1	9.0	8.5
Mirabegron + any antimuscarinic	788	28.6	2.7	2.6
Any ≥ 2 antimuscarinics (± mirabegron)	1847	67.0	6.3	6.0
Duloxetine + any OAB drug	144	5.2	0.5	0.5
Solifenacin + tolterodine	507	18.4	1.7	1.6
Oxybutynin + solifenacin	458	16.6	1.6	1.5
Mirabegron + solifenacin	417	15.1	1.4	1.4
Fesoterodine + solifenacin	174	6.3	0.6	0.6
Oxybutynin + tolterodine	174	6.3	0.6	0.6
Other combinations	1026	37.2	3.5	3.3

Drug class	N	% of all patient in OAB sub-cohort	% of all female patients
Any OAB drug	29,094	100	94.5
Any antimuscarinic	27,010	92.8	87.7
Total mirabegron	2972	10.2	9.7

*LUTS*: lower urinary tract symptoms; *OAB*: overactive bladder

#### Male LUTS (including OAB) sub-cohort

In this sub-cohort of 12,383 men treated with any OAB drug, 64.2% received monotherapy with an OAB drug and 35.8% received combinations (31.2% with any OAB drug plus any BPO drug and 7.8% with two or more OAB drugs [3.2% with and 4.6% without a BPO drug]) (Table 3). In addition, 94.1% received any antimuscarinic agent, 8.4% received mirabegron, 2.5% received any antimuscarinic agent plus mirabegron and 5.4% received two or more antimuscarinic agents (Table 3). In this sub-cohort 26.9% of patients received combination therapy including any alpha-blocker and any antimuscarinic agent and 2.6% received any alpha-blocker plus mirabegron. Furthermore, 7.7% of this sub-cohort received triple therapy with any alpha-blocker plus any 5-ARI plus any OAB drug (Table 3).

The most frequently prescribed OAB monotherapies in this sub-cohort were solifenacin (22.3%), oxybutynin (21.1%), tolterodine (11.7%) and mirabegron (4.1%) (Table 3; Additional file 1: Table S5).

The most common OAB drug either alone or in combination was solifenacin (40.6% of the OAB sub-cohort). The most frequently prescribed combination was solifenacin plus tamsulosin (7.3% of sub-cohort for this two-drug combination alone; 11.7% when also including additional drugs), followed by oxybutynin plus tamsulosin and then tolterodine plus tamsulosin, with 2.9% and 2.0% of sub-cohort, respectively. A total of 0.8% of this sub-cohort received mirabegron plus solifenacin alone (1.4% when also including additional drugs).

#### Male BPO sub-cohort

In this sub-cohort of 36,307 men, 81.9% received monotherapy (75.6% with any alpha-blocker; 6.3% with any 5-ARI) and 18.1% received combination therapy (15.3% with any alpha-blocker plus any 5-ARI). Tamsulosin monotherapy was the most commonly prescribed (58.3% of sub-cohort patients), followed by doxazosin (15.0%) (Table 4; Additional file 1: Table S6). For combination therapy, 49.7% of combination therapy patients were prescribed finasteride plus tamsulosin (9.0% of this sub-cohort for these two drugs alone; 10.0% when also including additional drugs), followed by dutasteride plus tamsulosin and doxazosin plus tamsulosin*.* In addition, 93.6% of patients in this sub-cohort overall received an alpha-blocker and 21.7% a 5-ARI.

#### Female LUTS (including OAB) sub-cohort

In this sub-cohort of 29,094 women, 90.5% of patients received monotherapy (83.4% any antimuscarinic, 7.1% mirabegron) and 9.5% combination therapy (9.0% with two or more OAB drugs). In addition, 0.5% of women in this sub-cohort received any OAB drug plus duloxetine (Table 5). Mirabegron plus duloxetine was prescribed to 0.04% of patients in this sub-cohort. A combination of any two or more antimuscarinic agents (± mirabegron) was used by 6.3% of sub-cohort patients and any antimuscarinic agent plus mirabegron by 2.7%. The most common OAB drug either alone or in combination was solifenacin (41.0% of the OAB sub-cohort) and 10.2% of this sub-cohort overall received mirabegron.

Solifenacin was the most frequently prescribed monotherapy (34.7% of this sub-cohort), followed by oxybutynin and tolterodine (Table 5; Additional file 1: Table S7); 92.8% of sub-cohort patients were prescribed any antimuscarinic agent. The most frequently prescribed combinations were solifenacin plus tolterodine, oxybutynin plus solifenacin and mirabegron plus solifenacin.

#### Female SUI sub-cohort

Duloxetine was prescribed to all (*n* = 1688) sub-cohort patients and was the only drug used in women with SUI, as defined in the base-case.

### Persistence

Kaplan Meier curves of TTD by mono- and combination drug therapy for the male BPO sub-cohort, and for duloxetine in the female SUI sub-cohort, are included in Additional file 1: Figures S1–S3.

#### Male LUTS (including OAB) sub-cohort

**Fig. 2:**
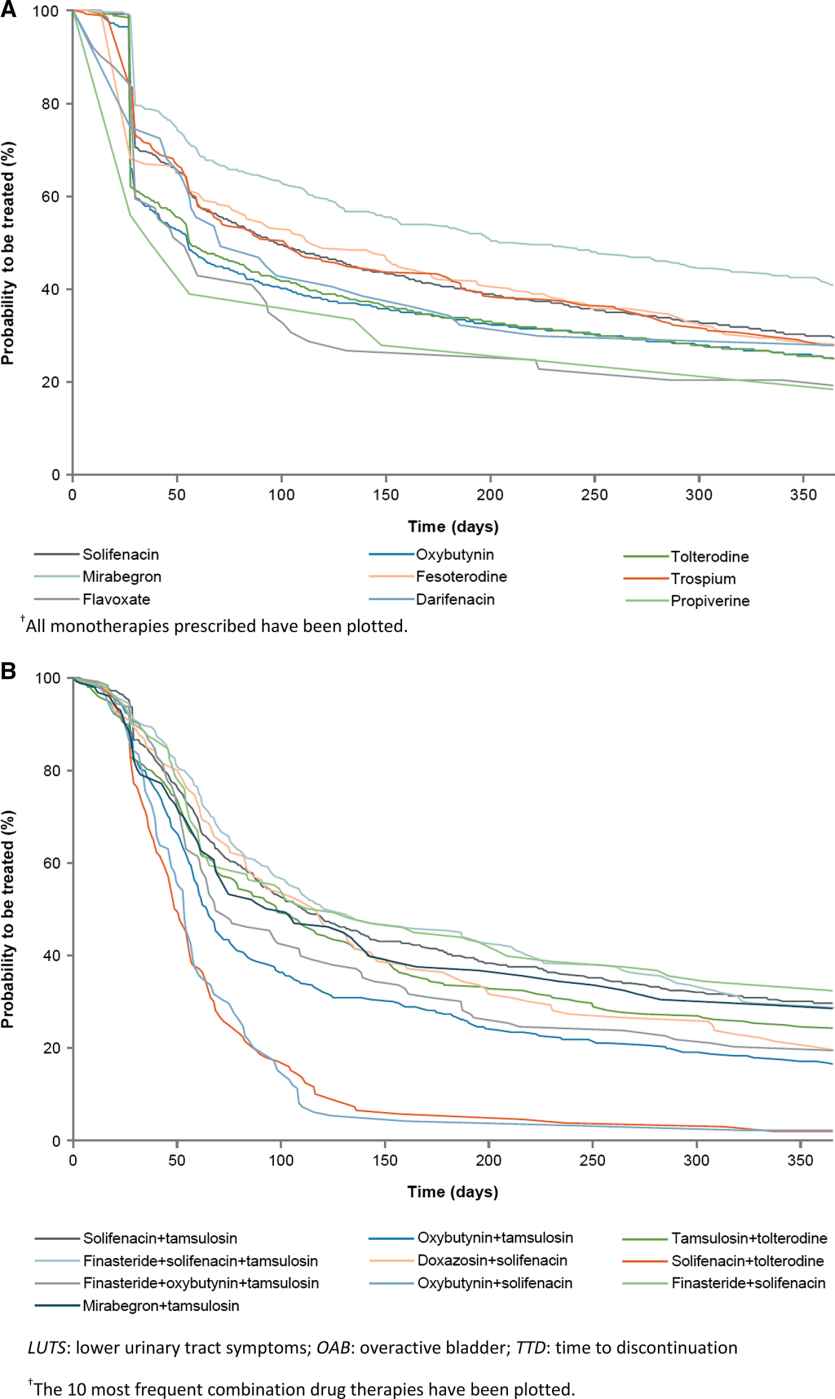
TTD in male LUTS (including OAB) sub-cohort (Kaplan–Meier estimates)^†^ for **A** monotherapies. ^†^All monotherapies prescribed have been plotted. **B** Combination drug therapies. *LUTS*: lower urinary tract symptoms; *OAB*: overactive bladder; *TTD*: time to discontinuation. ^†^The 10 most frequent combination drug therapies have been plotted

For monotherapy, mirabegron had the longest median TTD (205 days), followed by fesoterodine (115 days), trospium (102 days) and solifenacin (97 days) (Fig. 2a; Table 6). These drugs also displayed the highest 12-month persistence rates (Table 7). For combination therapies, tamsulosin plus trospium had the longest median TTD (144 days); however, the sample size was small (*n* = 65). The longest median TTD for combination therapy with a sample size > 100 was 121 days for finasteride plus solifenacin plus tamsulosin (Fig. 2b). The highest 12-month persistence was with finasteride plus solifenacin (32.3%) (Table 7).

**Table 6:** Persistence in post-index period in the male LUTS (including OAB) sub-cohort

Index drug	N	Persistence days Median (Q1–Q3)
*Monotherapy*		
Solifenacin	2759	97 (30–365^a^)
Oxybutynin	2613	56 (28–363)
Tolterodine	1445	56 (28–359)
Mirabegron	503	205 (50–365^a^)
Fesoterodine	285	115 (28–365^a^)
Trospium	227	102 (30–365^a^)
Flavoxate	49	54 (30–222)
Darifenacin	47	71 (28–365^a^)
Propiverine	18	42 (28–273)
*Combination drug therapy*		
Solifenacin + tamsulosin	902	111 (53–365^a^)
Oxybutynin + tamsulosin	364	65 (42–195)
Tamsulosin + tolterodine	248	99 (48–335.5)
Finasteride + solifenacin + tamsulosin	233	121 (61–365^a^)
Doxazosin + solifenacin	114	117 (59–308)
Solifenacin + tolterodine	114	50 (34–75)
Finasteride + oxybutynin + tamsulosin	111	69 (49–215)
Oxybutynin + solifenacin	102	54 (38–82)
Finasteride + solifenacin	96	120 (55–365^a^)
Mirabegron + tamsulosin	96	98 (47–365^a^)
Doxazosin + oxybutynin	95	60 (44–128)
Mirabegron + solifenacin	95	61 (42–95)
Fesoterodine + tamsulosin	83	80 (50–221)
Dutasteride + solifenacin + tamsulosin	79	74 (51–235)
Doxazosin + tolterodine	69	95 (50–365^a^)
Tamsulosin + trospium	65	144 (61–365^a^)
Finasteride + oxybutynin	62	77 (42–240)
Finasteride + tamsulosin + tolterodine	57	119 (62–365^a^)
Alfuzosin + solifenacin	47	124 (42–365^a^)
Other combinations	1405	63 (41–151)

*LUTS*: lower urinary tract symptoms; *OAB*: overactive bladder; *Q1*: lower quartile; *Q3*: upper quartile

^a^Q3 not reached by 365 days

**Table 7:** Persistence at 1 month, 6 months and 1 year in male LUTS (including OAB) sub-cohort

	N	1 month % [95% CI]	6 months % [95% CI]	1 year % [95% CI]	Median (months) [95% CI]
*All*	12,383	72.9	34.8	25.2	2.5
		[72.1, 73.6]	[33.9, 35.6]	[24.4, 26.0]	[2.4, 2.7]
Number of patients still at risk^a^		9024	4304	3105	
*Monotherapy*	7946	65.6	37.6	27.7	2.5
		[64.5, 66.6]	[36.5, 38.6]	[26.8, 28.7]	[2.3, 2.7]
Number of patients still at risk^a^		5211	2986	2193	
*Combination drug therapy*	4437	85.9	29.7	20.6	2.5
		[84.9, 86.9]	[28.4, 31.1]	[19.4, 21.8]	[2.4, 2.7]
Number of patients still at risk^a^		3813	1318	912	
*Monotherapy*					
*Darifenacin*	47	74.5	34.0	27.7	2.3
		[59.4, 84.6]	[21.0, 47.5]	[15.9, 40.8]	[1.7, 5.4]
Number of patients still at risk^a^		35	16	13	
*Fesoterodine*	285	67.4	41.8	28.1	3.8
		[61.6, 72.5]	[36.0, 47.4]	[23.0, 33.4]	[2.7, 5.2]
Number of patients still at risk^a^		192	119	80	
*Flavoxate*	49	59.2	26.5	20.4	1.8
		[44.2, 71.4]	[15.2, 39.3]	[10.5, 32.6]	[1.0, 3.1]
Number of patients still at risk^a^		29	13	9	
*Mirabegron*	503	79.5	53.1	40.9	6.7
		[75.7, 82.8]	[48.6, 57.3]	[36.6, 45.2]	[5.2, 9.5]
Number of patients still at risk^a^		400	267	204	
*Oxybutynin*	2613	59.6	33.5	25.1	1.8
		[57.7, 61.4]	[31.7, 35.3]	[23.5, 26.8]	[1.7, 1.9]
Number of patients still at risk^a^		1557	875	653	
*Propiverine*	18	55.6	27.8	22.2	1.4
		[30.5, 74.8]	[10.1, 48.9]	[6.9, 42.9]	[0.9, 4.9]
*Number of patients still at risk*^a^		10	5	4	
*Solifenacin*	2759	70.2	40.2	29.5	3.2
		[68.5, 71.9]	[38.4, 42.1]	[27.8, 31.2]	[2.9, 3.6]
Number of patients still at risk^a^		1938	1110	812	
*Tolterodine*	1445	61.2	33.7	24.8	1.8
		[58.6, 63.6]	[31.3, 36.1]	[22.6, 27.1]	[1.8, 2.2]
Number of patients still at risk^a^		884	487	355	
*Trospium*	227	73.1	41.4	27.8	3.4
		[66.9, 78.4]	[35.0, 47.7]	[22.1, 33.7]	[2.2, 4.9]
Number of patients still at risk^a^		166	94	63	
*Combination drug therapy*					
*Alfuzosin* + *solifenacin*	47	87.2	40.4	29.8	4.1
		[73.8, 94.1]	[26.5, 53.9]	[17.6, 43.0]	[2.1, 6.9]
Number of patients still at risk^a^		41	19	14	
*Doxazosin* + *oxybutynin*	95	87.4	18.9	NO	2
		[78.8, 92.6]	[11.8, 27.4]	–	[1.8, 2.3]
Number of patients still at risk^a^		83	18	13	
*Doxazosin* + *solifenacin*	114	89.5	36.0	20.2	3.8
		[82.2, 93.9]	[27.3, 44.7]	[13.4, 28.0]	[2.8, 4.7]
Number of patients still at risk^a^		102	41	23	
*Doxazosin* + *tolterodine*	69	87.0	39.1	29.0	3.1
		[76.4, 93.0]	[27.7, 50.4]	[18.8, 39.9]	[2.1, 6.4]
Number of patients still at risk^a^		60	27	20	
*Dutasteride* + *solifenacin* + *tamsulosin*	79	89.9	27.8	21.5	2.4
		[80.8, 94.8]	[18.5, 38.0]	[13.3, 31.1]	[2.1, 3.6]
Number of patients still at risk^a^		71	22	17	
*Fesoterodine* + *tamsulosin*	83	85.5	28.9	NO	2.6
		[75.9, 91.5]	[19.6, 38.9]	–	[2.1, 3.3]
Number of patients still at risk^a^		71	24	12	
*Finasteride* + *oxybutynin*	62	88.7	32.3	17.7	2.5
		[77.8, 94.5]	[21.1, 43.9]	[9.5, 28.1]	[1.8, 3.6]
Number of patients still at risk^a^		55	20	11	
*Finasteride* + *oxybutynin* + *tamsulosin*	111	91.0	30.6	19.8	2.3
		[83.9, 95.0]	[22.3, 39.3]	[13.0, 27.7]	[2.0, 3.6]
Number of patients still at risk^a^		101	34	22	
*Finasteride* + *solifenacin*	96	90.6	44.8	32.3	3.9
		[82.8, 95.0]	[34.7, 54.4]	[23.2, 41.7]	[2.1, 6.9]
Number of patients still at risk^a^		87	43	31	
*Finasteride* + *solifenacin* + *tamsulosin*	233	91.0	45.1	28.8	4
		[86.5, 94.0]	[38.6, 51.3]	[23.1, 34.7]	[3.4, 6.2]
Number of patients still at risk^a^		212	105	67	
*Finasteride* + *tamsulosin* + *tolterodine*	57	96.5	40.4	28.1	3.9
		[86.7, 99.1]	[27.7, 52.7]	[17.2, 40.0]	[2.8, 6.7]
Number of patients still at risk^a^		55	23	15	
*Finasteride* + *tolterodine*	41	85.4	29.3	22.0	1.7
		[70.3, 93.1]	[16.4, 43.4]	[10.9, 35.5]	[1.5, 3.9]
Number of patients still at risk^a^		35	12	9	
*Mirabegron* + *solifenacin*	95	85.3	NO	NO	2
		[76.4, 91.0]	–	–	[1.7, 2.5]
Number of patients still at risk^a^		81	11	6	
*Mirabegron* + *tamsulosin*	96	82.3	37.5	29.2	3.2
		[73.1, 88.6]	[27.9, 47.1]	[20.5, 38.4]	[2.2, 4.7]
Number of patients still at risk^a^		79	36	28	
*Oxybutynin* + *solifenacin*	102	84.3	NO	NO	1.8
		[75.7, 90.1]	–	–	[1.6, 1.9]
Number of patients still at risk^a^		86	4	2	
*Oxybutynin* + *tamsulosin*	364	82.4	26.9	16.5	2.1
		[78.1, 86.0]	[22.5, 31.6]	[12.9, 20.5]	[2.0, 2.3]
Number of patients still at risk^a^		300	98	60	
*Solifenacin* + *tamsulosin*	902	86.7	39.9	29.4	3.6
		[84.3, 88.7]	[36.7, 43.1]	[26.4, 32.4]	[3.2, 4.1]
Number of patients still at risk^a^		782	360	264	
*Solifenacin* + *tolterodine*	114	77.2	NO	NO	1.6
		[68.3, 83.9]	–	–	[1.5, 1.8]
Number of patients still at risk^a^		88	6	2	
*Tamsulosin* + *tolterodine*	248	82.3	33.5	24.2	3.3
		[76.9, 86.5]	[27.7, 39.4]	[19.1, 29.7]	[2.6, 4.0]
Number of patients still at risk^a^		204	83	60	
*Tamsulosin* + *trospium*	65	90.8	41.5	29.2	4.7
		[80.6, 95.7]	[29.5, 53.1]	[18.8, 40.5]	[2.9, 7.1]
Number of patients still at risk^a^		59	27	19	
*Other combinations*	1364	85.1	22.4	15.9	2.1
		[83.1, 86.9]	[20.2, 24.6]	[14.0, 17.9]	[2.0, 2.2]
Number of patients still at risk^a^		1161	305	217	

Not Observable indicates that the number of patients still at risk was below the 20% of the initial sample threshold required to calculate persistence, or the median was not reached

*CI*: confidence interval; *LUTS*: lower urinary tract symptoms; *NO*: not observable; *OAB*: overactive bladder ^a^Number of patients still observable at a given time and for whom no events occurred

#### Male BPO sub-cohort

For monotherapy, median TTD was longest for doxazosin and finasteride (> 365 days each [median not reached]), followed by tamsulosin (329 days) and dutasteride (305 days) (however, see sensitivity analyses below). Doxazosin had the highest 12-month persistence (67.8%) followed by finasteride (53.3%) and tamsulosin (48.4%). For combination therapies, the highest 12-month persistence was for dutasteride plus tamsulosin (56.3%) followed by doxazosin plus dutasteride (55.6%) (Additional file 1: Tables S8 and S9).

#### Female LUTS (including OAB) sub-cohort

**Fig. 3:**
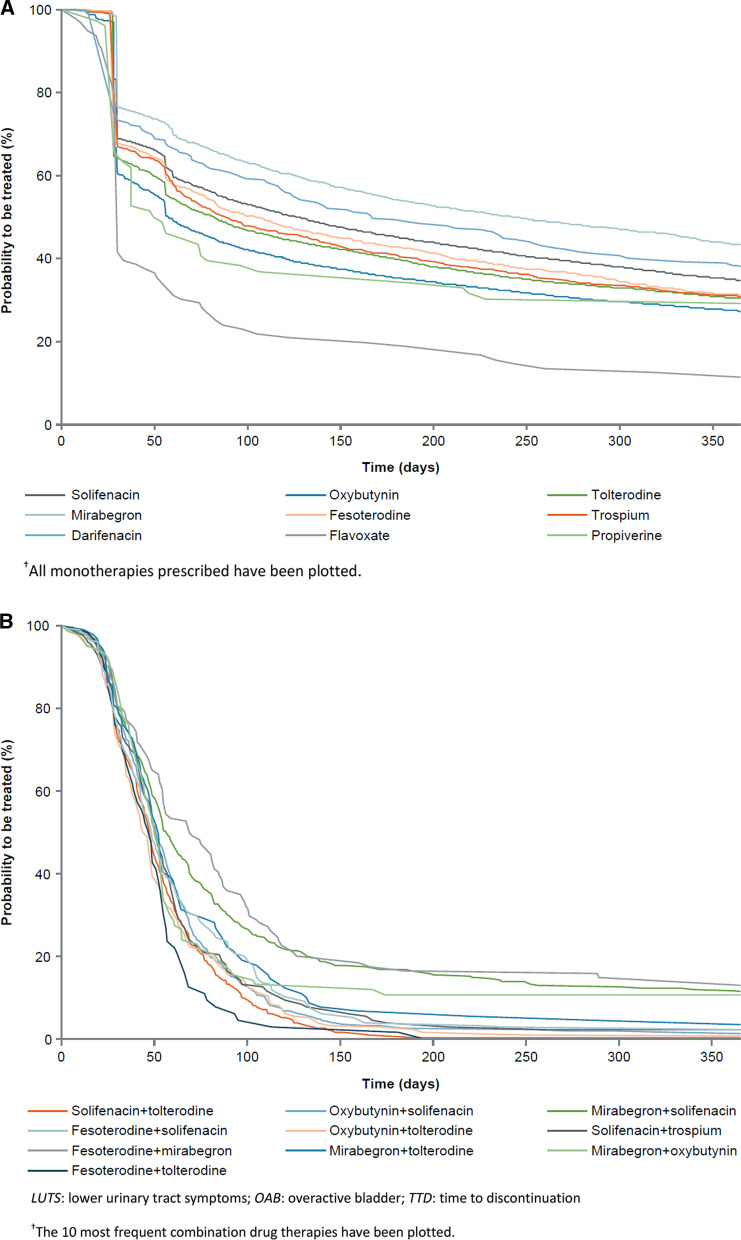
TTD in female LUTS (including OAB) sub-cohort (Kaplan–Meier estimates)^†^for **A** monotherapies. ^†^All monotherapies prescribed have been plotted. **B** Combination drug therapies. *LUTS*: lower urinary tract symptoms; *OAB*: overactive bladder; *TTD*: time to discontinuation. ^†^The 10 most frequent combination drug therapies have been plotted

For monotherapy, median TTD (244 days) and 12-month persistence (43.5%) was longest for mirabegron (Fig. 3a). Among combination therapies, persistence rates at 12 months were often not observable; however, the longest median TTD was 69 days for duloxetine plus tolterodine and 70 days for fesoterodine plus mirabegron (Fig. 3b, and Additional file 1: Tables S10 and S11)*.*

#### Female SUI sub-cohort

For duloxetine, median TTD was 55 days and 12-month persistence rate was 22.0%.

### Sensitivity analyses

Results of sensitivity analyses were consistent with the main analyses, with one notable exception. In the male BPO sub-cohort, patients with a confirmed LUTS diagnosis had a lower median TTD on doxazosin (144 versus > 365 days) and finasteride (170 versus > 365 days) versus the main analysis (Additional file 1: Figures S4a and b). There were no notable findings in the other sensitivity analyses performed (Additional file 1: Table S12).

## Discussion

This retrospective analysis in a UK GP primary care database complements previous UK studies of LUTS/OAB and LUTS/BPO [20–22]. The study highlights the relatively low use of combination treatments that target OAB. Only a small proportion of LUTS (including OAB) patients were prescribed mirabegron with an antimuscarinic agent. However, use of this combination may have increased since the publication of studies such as BESIDE in 2016, SYNERGY in 2017 and SYNERGY II in 2018, which showed benefits of combination versus an antimuscarinic alone [9, 23, 24]. It was also notable that around 5% each of men and women receiving OAB drugs were on a combination of two or more antimuscarinic agents (Tables 3 and 4), despite a lack of evidence supporting any benefit of this approach.

Our study also highlights the relatively low treatment rates for storage symptoms in men, despite the fact that these symptoms can be highly bothersome in men, even more so than voiding symptoms [25]. In the EPIC study, of all men identified with LUTS, over 80% had storage LUTS [10]. In the EpiLUTS study [13], storage symptoms were experienced by around two-thirds of men, with approximately 50% of men reporting mixed storage and voiding symptoms [13]. Alpha-blockers are the usual first-line treatment for men with LUTS suggestive of BPO [6, 26] and while they have been shown to relieve both voiding and storage symptoms [27], evidence suggests that up to two-thirds will not respond adequately to alpha-blocker monotherapy [14]. In these cases, the EAU guidelines recommend adding an OAB drug [6]. Therefore, it would be expected that the percentage of men who required OAB treatment would be similar to the percentage of those with storage symptoms. This percentage is around 45% when calculated using data from the EpiLUTS study [13]—the percentage of LUTS patients with storage symptoms alone combined with around two thirds of LUTS patients with mixed symptoms (i.e., those who do not respond to first-line alpha-blocker therapy). However, in our study only around a quarter of all men being treated for LUTS received treatment specifically targeting storage symptoms (i.e., an antimuscarinic and/or mirabegron alone or combined with a BPO drug), although this figure refers only to men with treated LUTS, in contrast to the other studies which were based on the general population. Thus, some men with storage symptoms may be receiving inadequate treatment in clinical practice, despite storage symptoms often being the most bothersome component of LUTS [28].

We might also expect to see OAB/BPO drug combination therapy in up to one-third of all men being treated for LUTS (i.e., two-thirds of those with mixed symptoms) [13, 14]. However, in the current study, only 7% were receiving alpha-blocker plus antimuscarinic combination treatment therapy (and only 8% were on any OAB/BPO drug combination), which is consistent with another UK study in which 15% of men with mixed LUTS were reported to be receiving an alpha-blocker combined with an antimuscarinic agent [20].

As well as alpha-blockers and 5-ARIs, the EAU guidelines recommend the use of phosphodiesterase type 5 inhibitors (PDE5Is) for the treatment of men with moderate-to-severe LUTS with or without erectile dysfunction [6]. Tadalafil, the only currently licensed PDE5I for male LUTS [6], has been shown to relieve both voiding and storage symptoms [29], although additional therapy may be required for patients with severe LUTS related to BOO [30]. However, this study did not look at tadalafil use due to potential misclassification of patients receiving the drug for erectile dysfunction.

The reasons for the low treatment of storage symptoms in men may be historical, reflecting overemphasis on the prostate-related component of LUTS rather than bladder-related issues. Furthermore, there may be a perceived risk of precipitating urinary retention when using bladder antimuscarinic agents in men with evidence of obstruction, although the available evidence suggests that this risk is low [31]. There is already good evidence supporting the use of alpha-blocker/antimuscarinic combination therapy in men with mixed symptoms [16, 17]. More recently, two randomized, placebo-controlled trials have also demonstrated that mirabegron add-on therapy in men who have residual OAB symptoms while being treated with tamsulosin for LUTS is both effective and well-tolerated [18, 32]. It is hoped that this new evidence will help to improve the overall management of men with mixed symptoms.

Antimuscarinic agents and beta-3 agonists are recommended first-line pharmacological treatments for both men and women with OAB [7, 8] and men with moderate-to-severe LUTS with predominant bladder storage symptoms [6]. However, with antimuscarinics, long-term persistence is often poor due to unmet treatment expectations or adverse events [33]. In our study, mirabegron monotherapy had the highest persistence (both in men and women). Several observational studies also reported higher persistence with mirabegron vs antimuscarinics [22, 34]. Persistence was greater with drug monotherapy than in combination drug therapy, and was particularly poor with combinations of two antimuscarinics in both men and women.

For monotherapy targeting BPO and voiding symptoms (e.g. alpha-blockers and 5-ARIs), persistence was highest for doxazosin and finasteride, but this was not evident in sensitivity analyses based on confirmed LUTS diagnosis. This suggests that the higher persistence with these agents in the main sub-cohorts may be driven by their use in other disorders (e.g., doxazosin for hypertension) and it is notable that only 3% of patients on doxazosin had a LUTS diagnostic code.

A limitation of our study is that in CPRD GOLD, GPs do not systematically report prescriptions issued in secondary care, and reasons for discontinuation were not available in CPRD, which limits interpretation of persistence results. In addition, some treatments are prescribed for conditions other than OAB, LUTS, BPO or SUI (e.g., doxazosin, finasteride and duloxetine), which may influence some of the treatment pattern and/or persistence estimates. The inclusion of fixed-dose combinations may increase the overall persistence with tamsulosin/solifenacin combination therapy [35]; for tamsulosin/dutasteride fixed-dose combination, the available evidence suggests it may have no impact on persistence [36]. Finally, as this study was performed using a UK general practice database, it is unclear to what extent the results would be generalizable to other healthcare systems.

## Conclusions

This study provides new real-world evidence suggesting that men with LUTS may be under-treated with pharmacotherapies that specifically target storage symptoms. Only around a quarter of the men being treated for any LUTS received treatment specifically targeting storage symptoms and around 8% of men received a combination of BPO/OAB drugs for mixed symptoms. In addition, use of combination OAB treatment was low in both men and women, which may reflect the lack of evidence for this approach at the time these patients were being treated. Of all OAB medications, numerically the highest rates of monotherapy persistence were seen with mirabegron in both men and women. Persistence was worse when using combination drug therapy and particularly poor when using two antimuscarinic agents. By highlighting the possible under-treatment of men with treatments that target storage symptoms and the low use of combination OAB treatment (especially with mirabegron plus an antimuscarinic agent), this may help clinicians in the UK to re-assess their approach to pharmacotherapy for patients with bothersome LUTS.

## Supplementary Information

**Additional file 1.** Supplementary Tables 1 to 12 and Supplementary Figures 1 to 4.

## Data Availability

Researchers may request access to anonymized participant level data, trial level data and protocols from Astellas sponsored clinical trials at www.clinicalstudydatarequest.com. For the Astellas criteria on data sharing see: https://clinicalstudydatarequest.com/Study-Sponsors/Study-Sponsors-Astellas.aspx.

## Abbreviations

5-ARI: 5-Alpha reductase inhibitor
BNF: British National Formulary
BOO: Bladder outlet obstruction
BPO: Benign prostatic obstruction
CI: Confidence interval
CPRD: Clinical Practice Research Datalink
EAU: European Association of Urology
GP: General practitioner
IMD: Index of Multiple Deprivation
LUTS: Lower urinary tract symptoms
MAGD: Maximum allowable gap duration
NO: Not observable
NRES: National Research Ethics Service Committee
OAB: Overactive bladder
PDE5I: Phosphodiesterase type 5 inhibitor
Q1: Lower quartile
Q3: Upper quartile
QOF: Quality and Outcomes Framework
SA: Sensitivity analysis
SD: Standard deviation
SUI: Stress urinary incontinence
TTD: Time to discontinuation
