# Supplementary material for: The use of mono- and combination drug therapy in men and women with lower urinary tract symptoms (LUTS) in the UK: a retrospective observational study
Source: BMC Urol. 2021 Sep 2;21:119. doi: 10.1186/s12894-021-00881-w (PMC8414666; doi:10.1186/s12894-021-00881-w)
Supplement: Supplementary file 1 — Additional file 1. Supplementary Tables 1 to 12 and Supplementary Figures 1 to 4. [file 12894_2021_881_MOESM1_ESM.docx]

**The use of mono- and combination drug therapy in men and women with lower urinary tract symptoms (LUTS) in the UK: a retrospective observational study**

Mahmood Ali, et al

**Supplementary Information**

Contents

[Supplementary Table 1. Drugs of interest in male and female cohorts 2](#_Toc74747539)

[Supplementary Table 2a. Read codes for LUTS symptoms 3](#_Toc74747540)

[Supplementary Table 2b. Read codes for OAB and storage LUTS [1] 3](#_Toc74747541)

[Supplementary Table 2c. Read codes for voiding LUTS, prostatism, and storage LUTS [1] 4](#_Toc74747542)

[Supplementary Table 3a. Distribution of the male cohort by sub-cohorts 6](#_Toc74747543)

[Supplementary Table 3b. Distribution of the female cohort by sub-cohorts 6](#_Toc74747544)

[Supplementary Table 4. Extent of drug class use in all men (N = 48690) 7](#_Toc74747545)

[Supplementary Table 5. Extent of mono- and combination drug therapy use in the male LUTS (including OAB) sub-cohort 8](#_Toc74747546)

[Supplementary Table 6. Extent of mono- and combination drug therapy use in the male BPO sub-cohort 10](#_Toc74747547)

[Supplementary Table 7. Extent of mono- and combination drug therapy use in the female LUTS (including OAB) sub-cohort 12](#_Toc74747548)

[Supplementary Table 8. Persistence in post-index period in the male BPO sub-cohort 14](#_Toc74747549)

[Supplementary Table 9. Persistence at 1 month, 6 months and 1 year in the male BPO population 15](#_Toc74747550)

[Supplementary Table 10. Persistence in post-index period in the female LUTS (including OAB) sub-cohort 19](#_Toc74747551)

[Supplementary Table 11. Persistence at 1 month, 6 months and 1 year in female LUTS (including OAB) population 20](#_Toc74747552)

[Supplementary Table 12. Extent of mono- and combination drug therapy use in the male BPO sub-cohort - Sensitivity analyses 25](#_Toc74747553)

[Supplementary Figure 1. TTD for monotherapies in male BPO sub-cohort (Kaplan-Meier estimates) 27](#_Toc74747554)

[Supplementary Figure 2. TTD for combinations in male BPO sub-cohort (Kaplan-Meier estimates)^†^ 28](#_Toc74747555)

[Supplementary Figure 3. TTD for duloxetine in female SUI sub-cohort (Kaplan-Meier estimates) 29](#_Toc74747556)

[Supplementary Figure 4. Kaplan-Meier curves for TTD sensitivity analysis: (A) monotherapy; (B) combination drug therapy (male BPO/LUTS population^†^) 30](#_Toc74747557)

# Supplementary Table 1. Drugs of interest in male and female cohorts

| **Male cohort:** | | **Female cohort:** | |
| --- | --- | --- | --- |
| **Drug class** | **Drugs** | **Drug class** | **Drugs** |
| **LUTS (incl. OAB)** | | **LUTS (incl. OAB)** | |
| Antimuscarinics | darifenacin, flavoxate, fesoterodine, oxybutynin, propiverine, solifenacin*, tolterodine and trospium | Antimuscarinics | darifenacin, flavoxate, fesoterodine, oxybutynin, propiverine, solifenacin, tolterodine and trospium |
| Beta(3)-adrenoceptor agonists | mirabegron | Beta(3)-adrenoceptor agonists | mirabegron |
| **BPO** | | **SUI** | |
| Alpha-blockers | alfuzosin, doxazosin, indoramin, prazosin, tamsulosin*, terazosin | Serotonin and noradrenaline re-uptake inhibitors | duloxetine |
| 5-alpha reductase inhibitors | finasteride, dutasteride* | – | – |
| Fixed-dose combinations | tamsulosin + solifenacin,  tamsulosin + dutasteride | – | – |

*BPO*: benign prostatic obstruction; *LUTS*: lower urinary tract symptoms; *OAB*: overactive bladder; *SUI*: stress urinary incontinence

The fixed-dose combinations are products Vesomni™ (tamsulosin + solifenacin), Combodart® (tamsulosin + dutasteride); *Including fixed-dose combination products Vesomni™, Combodart®.

# Supplementary Table 2a. Read codes for LUTS symptoms

| **Diagnosis** | **medcode** | **readcode** | **readterm** |
| --- | --- | --- | --- |
| LUTS | 99784 | 1AZ6.00 | Lower urinary tract symptoms |
| LUTS | 104539 | 1AZ6000 | Mild lower urinary tract symptoms |
| LUTS | 104390 | 1AZ6100 | Moderate lower urinary tract symptoms |
| LUTS | 104344 | 1AZ6200 | Severe lower urinary tract symptoms |

*LUTS*: lower urinary tract symptoms

# Supplementary Table 2b. Read codes for OAB and storage LUTS [1]

| **Diagnosis** | **medcode** | **readcode** | **readterm** |
| --- | --- | --- | --- |
| OAB & storage LUTS | 6724 | 1A1..00 | Micturition frequency |
| OAB & storage LUTS | 4160 | 1A1..11 | Frequency of micturition |
| OAB & storage LUTS | 729 | 1A1..13 | Urinary frequency |
| OAB & storage LUTS | 6558 | 1A2..00 | Micturition control |
| OAB & storage LUTS | 17567 | 1A2..11 | Urinary control |
| OAB & storage LUTS | 5959 | 1A25.00 | Urgency |
| OAB & storage LUTS | 583 | 1A25.11 | Urgency of micturition |
| OAB & storage LUTS | 3887 | 1A26.00 | Urge incontinence of urine |
| OAB & storage LUTS | 12289 | K16..00 | Other disorders of bladder |
| OAB & storage LUTS | 6676 | K165300 | Detrusor instability |
| OAB & storage LUTS | 5993 | K165400 | Unstable bladder |
| OAB & storage LUTS | 25836 | K165z00 | Other bladder function disorder NOS |
| OAB & storage LUTS | 887 | K16y400 | Irritable bladder |
| OAB & storage LUTS | 2248 | K16y411 | Detrusor instability |
| OAB & storage LUTS | 31318 | K16y412 | Unstable bladder |
| OAB & storage LUTS | 33913 | K16z.00 | Bladder disorders NOS |
| OAB & storage LUTS | 108426 | Kyu5200 | [X] Other neuromuscular dysfunction of bladder |
| OAB & storage LUTS | 65634 | Kyu5300 | [X] Other specified disorders of bladder |
| OAB & storage LUTS | 90866 | Kyu5E00 | [X] Neuromuscular dysfunction of bladder, unspecified |
| OAB & storage LUTS | 98627 | Kyu5F00 | [X] Urethral disorder, unspecified |
| OAB & storage LUTS | 17320 | R083200 | [D] Urge incontinence |
| OAB & storage LUTS | 503 | R084000 | [D] Frequency of micturition, unspecified |

*LUTS*: lower urinary tract symptoms; *NOS*: not otherwise specified; *OAB*: overactive bladder

# Supplementary Table 2c. Read codes for voiding LUTS, prostatism, and storage LUTS [1]

| **Diagnosis** | **medcode** | **readcode** | **readterm** |
| --- | --- | --- | --- |
| Voiding LUTS, prostatism, and storage LUTS | 9589 | 1A3..00 | Micturition stream |
| Voiding LUTS, prostatism, and storage LUTS | 7027 | 1A3..11 | Urine stream |
| Voiding LUTS, prostatism, and storage LUTS | 9274 | 1A33.00 | Micturition stream poor |
| Voiding LUTS, prostatism, and storage LUTS | 25649 | 1A34.00 | Hesitancy |
| Voiding LUTS, prostatism, and storage LUTS | 7487 | 1A34.11 | Hesitancy of micturition |
| Voiding LUTS, prostatism, and storage LUTS | 42735 | 1A35.11 | Precipitancy of micturition |
| Voiding LUTS, prostatism, and storage LUTS | 2756 | 1A36.00 | Terminal dribbling of urine |
| Voiding LUTS, prostatism, and storage LUTS | 5705 | 1A37.00 | Dribbling of urine |
| Voiding LUTS, prostatism, and storage LUTS | 36366 | 1A3Z.00 | Micturition stream NOS |
| Voiding LUTS, prostatism, and storage LUTS | 5906 | 1AA..00 | Prostatism |
| Voiding LUTS, prostatism, and storage LUTS | 6938 | K160.13 | BOO - Bladder outflow obstruction |
| Voiding LUTS, prostatism, and storage LUTS | 16921 | K165200 | Bladder outflow obstruction |
| Voiding LUTS, prostatism, and storage LUTS | 3045 | K20..00 | Benign prostatic hypertrophy |
| Voiding LUTS, prostatism, and storage LUTS | 15346 | K20..11 | Benign adenoma of prostate |
| Voiding LUTS, prostatism, and storage LUTS | 25711 | K20..12 | Benign fibroma of prostate |
| Voiding LUTS, prostatism, and storage LUTS | 71354 | K20..13 | Benign myoma of prostate |
| Voiding LUTS, prostatism, and storage LUTS | 7702 | K20..14 | Enlarged prostate - benign |
| Voiding LUTS, prostatism, and storage LUTS | 7555 | K20..15 | BPH - benign prostatic hypertrophy |
| Voiding LUTS, prostatism, and storage LUTS | 2627 | K20..16 | Prostatism |
| Voiding LUTS, prostatism, and storage LUTS | 929 | K200.00 | Prostatic hyperplasia unspecified |
| Voiding LUTS, prostatism, and storage LUTS | 64296 | K201.00 | Prostatic hyperplasia of the lateral lobe |
| Voiding LUTS, prostatism, and storage LUTS | 35676 | K202.00 | Prostatic hyperplasia of the medial lobe |
| Voiding LUTS, prostatism, and storage LUTS | 16035 | K20z.00 | Prostatic hyperplasia NOS |

*BOO*: bladder outlet obstruction; *BPH*: benign prostatic hyperplasia; *LUTS*: lower urinary tract symptoms; *NOS*: not otherwise specified

# Supplementary Table 3a. Distribution of the male cohort by sub-cohorts

|  | **N** | **%** |
| --- | --- | --- |
| **Male cohort** | **48690** | **100.0** |
| - **LUTS (including OAB) sub-cohort** | 12383 | 25.4 |
| *LUTS alone* | 8525 | 17.5 |
| - *Monotherapy* | 7946 | 16.3 |
| - *Combinations* | 579 | 1.2 |
| *LUTS + BPO* | 3858 | 7.9 |
| - **BPO sub-cohort (BPO alone)** | 36307 | 74.6 |
| - *Monotherapy* | 29739 | 61.1 |
| - *Combinations* | 6568 | 13.5 |

*BPO*: benign prostatic obstruction; *LUTS*: lower urinary tract symptoms; *OAB*: overactive bladder

# Supplementary Table 3b. Distribution of the female cohort by sub-cohorts

|  | **N** | **%** |
| --- | --- | --- |
| **Female cohort** | **30782** | **100.0** |
| - **LUTS including (OAB) sub-cohort** | 29094 | 94.5 |
| *OAB alone* | 28950 | 94.0 |
| - *Monotherapy* | 26338 | 85.6 |
| - *Combinations* | 2612 | 8.5 |
| *OAB + SUI* | 144 | 0.5 |
| - **SUI sub-cohort (SUI alone)** | 1688 | 5.5 |

*LUTS*: lower urinary tract symptoms; *OAB*: overactive bladder; *SUI*: stress urinary incontinence

# Supplementary Table 4. Extent of drug class use in all men (N = 48690)

| Drug class/drug class combination | N | % of male patients |
| --- | --- | --- |
| Any OAB drug | 12383 | 25.4 |
| Any antimuscarinic | 11653 | 23.9 |
| Total mirabegron | 1039 | 2.1 |
| Any BPO drug | 40170 | 82.5 |
| Any alpha-blocker | 37522 | 77.1 |
| Any 5-ARI | 9180 | 18.9 |
| Any BPO drug + any OAB drug | 3863 | 7.9 |
| Any alpha-blocker + any OAB drug | 3538 | 7.3 |
| Any alpha-blocker + any antimuscarinic | 3330 | 6.8 |
| Any alpha-blocker + mirabegron | 326 | 0.7 |
| Any alpha-blocker + any 5-ARI | 6531 | 13.4 |
| Mirabegron + any antimuscarinic (±BPO drug) | 307 | 0.6 |
| Any ≥ 2 OAB (±BPO drug) drug | 964 | 2.0 |
| Any ≥ 2 OAB (no BPO drug) drug | 574 | 1.2 |
| Any ≥ 2 antimuscarinics (±BPO drug and/or mirabegron) | 666 | 1.4 |
| Any ≥ 2 alpha-blocker | 1427 | 2.9 |
| Any ≥ 2 5-ARI | 210 | 0.4 |

*5-ARI*: 5-alpha reductase inhibitor; *BPO*: benign prostatic obstruction; *OAB*: overactive bladder

# Supplementary Table 5. Extent of mono- and combination drug therapy use in the male LUTS (including OAB) sub-cohort

| **Monotherapy** | **N** | **% of monotherapy patients** | **% of all patients** | **Presence of a LUTS diagnosis Read code**  **n (%)** |
| --- | --- | --- | --- | --- |
| All monotherapy | 7946 | 100.0 | 64.2 | 1610 (20.3) |
| Solifenacin | 2759 | 34.7 | 22.3 | 565 (20.5) |
| Oxybutynin | 2613 | 32.9 | 21.1 | 517 (19.8) |
| Tolterodine | 1445 | 18.2 | 11.7 | 280 (19.4) |
| Mirabegron | 503 | 6.3 | 4.1 | 111 (22.1) |
| Fesoterodine | 285 | 3.6 | 2.3 | 63 (22.1) |
| Trospium | 227 | 2.9 | 1.8 | 53 (23.3) |
| Flavoxate | 49 | 0.6 | 0.4 | 7 (14.3) |
| Darifenacin | 47 | 0.6 | 0.4 | 12 (25.5) |
| Propiverine | 18 | 0.2 | 0.1 | 2 (11.1) |
| **Combination drug therapy** | **N** | **% of combination drug therapy patients** | **% of all patients** | **Presence of a LUTS diagnosis Read code**  **n (%)** |
| All combination drug therapy | 4437 | 100.0 | 35.8 | 1088 (24.5) |
| Solifenacin + tamsulosin | 902 | 20.3 | 7.3 | 226 (25.1) |
| Oxybutynin + tamsulosin | 364 | 8.2 | 2.9 | 97 (26.6) |
| Tamsulosin + tolterodine | 248 | 5.6 | 2.0 | 56 (22.6) |
| Finasteride + solifenacin + tamsulosin | 233 | 5.3 | 1.9 | 65 (27.9) |
| Doxazosin + solifenacin | 114 | 2.6 | 0.9 | 27 (23.7) |
| Solifenacin + tolterodine | 114 | 2.6 | 0.9 | 20 (17.5) |
| Finasteride + oxybutynin + tamsulosin | 111 | 2.5 | 0.9 | 24 (21.6) |
| Oxybutynin + solifenacin | 102 | 2.3 | 0.8 | 20 (19.6) |
| Finasteride + solifenacin | 96 | 2.2 | 0.8 | 22 (22.9) |
| Mirabegron + tamsulosin | 96 | 2.2 | 0.8 | 23 (24.0) |
| Doxazosin + oxybutynin | 95 | 2.1 | 0.8 | 19 (20.0) |
| Mirabegron + solifenacin | 95 | 2.1 | 0.8 | 20 (21.1) |
| Fesoterodine + tamsulosin | 83 | 1.9 | 0.7 | 26 (31.3) |
| Dutasteride + solifenacin + tamsulosin | 79 | 1.8 | 0.6 | 24 (30.4) |
| Doxazosin + tolterodine | 69 | 1.6 | 0.6 | 12 (17.4) |
| Tamsulosin + trospium | 65 | 1.5 | 0.5 | 16 (24.6) |
| Finasteride + oxybutynin | 62 | 1.4 | 0.5 | 18 (29.0) |
| Finasteride + tamsulosin + tolterodine | 57 | 1.3 | 0.5 | 18 (31.6) |
| Alfuzosin + solifenacin | 47 | 1.1 | 0.4 | 11 (23.4) |
| Finasteride + tolterodine | 41 | 0.9 | 0.3 | 9 (22.0) |
| Other combinations | 1364 | 30.7 | 11.0 | 335 (24.6) |

*LUTS*: lower urinary tract symptoms; *OAB*: overactive bladder

# Supplementary Table 6. Extent of mono- and combination drug therapy use in the male BPO sub-cohort

| **Monotherapy** | **N** | **% of monotherapy patients** | **% of all patients** | **Presence of a LUTS diagnosis Read code**  **n (%)** |
| --- | --- | --- | --- | --- |
| All monotherapy | 29739 | 100.0 | 81.9 | 4973 (16.7) |
| Tamsulosin | 21158 | 71.1 | 58.3 | 4270 (20.2) |
| Doxazosin | 5456 | 18.3 | 15.0 | 164 (3.0) |
| Finasteride | 2131 | 7.2 | 5.9 | 344 (16.1) |
| Alfuzosin | 665 | 2.2 | 1.8 | 141 (21.2) |
| Dutasteride | 146 | 0.5 | 0.4 | 34 (23.3) |
| Prazosin | 101 | 0.3 | 0.3 | 2 (2.0) |
| Terazosin | 44 | 0.1 | 0.1 | 10 (22.7) |
| Indoramin | 38 | 0.1 | 0.1 | 8 (21.1) |
| **Combination drug therapy** | **N** | **% of combination drug therapy patients** | **% of all patients** | **Presence of a LUTS diagnosis Read code**  **n (%)** |
| All combination drug therapy | 6568 | 100.0 | 18.1 | 1399 (21.3) |
| Finasteride + tamsulosin | 3262 | 49.7 | 9.0 | 736 (22.6) |
| Dutasteride + tamsulosin | 1189 | 18.1 | 3.3 | 286 (24.1) |
| Doxazosin + tamsulosin | 614 | 9.3 | 1.7 | 101 (16.4) |
| Doxazosin + finasteride | 289 | 4.4 | 0.8 | 62 (21.5) |
| Alfuzosin + finasteride | 233 | 3.5 | 0.6 | 50 (21.5) |
| Alfuzosin + tamsulosin | 208 | 3.2 | 0.6 | 42 (20.2) |
| Doxazosin + finasteride + tamsulosin | 167 | 2.5 | 0.5 | 26 (15.6) |
| Dutasteride + finasteride + tamsulosin | 125 | 1.9 | 0.3 | 21 (16.8) |
| Doxazosin + dutasteride + tamsulosin | 55 | 0.8 | 0.2 | 9 (16.4) |
| Dutasteride + finasteride | 46 | 0.7 | <0.2 | 5 (10.9) |
| Alfuzosin + dutasteride + tamsulosin | 44 | 0.7 | <0.2 | 9 (20.5) |
| Alfuzosin + finasteride + tamsulosin | 41 | 0.6 | <0.2 | 6 (14.6) |
| Tamsulosin + terazosin | 29 | 0.4 | <0.2 | 3 (10.3) |
| Alfuzosin + dutasteride | 25 | 0.4 | <0.2 | 6 (24.0) |
| Finasteride + terazosin | 25 | 0.4 | <0.2 | 10 (40.0) |
| Prazosin + tamsulosin | 23 | 0.4 | <0.2 | 3 (13.0) |
| Alfuzosin + doxazosin | 20 | 0.3 | <0.2 | 2 (10.0) |
| Indoramin + tamsulosin | 19 | 0.3 | <0.2 | 2 (10.5) |
| Doxazosin + dutasteride | 18 | 0.3 | <0.1 | 2 (11.1) |
| Finasteride + indoramin | 15 | 0.2 | <0.1 | 4 (26.7) |
| Other combinations | 121 | 1.8 | 0.3 | 14 (11.6) |

*BPO*: benign prostatic obstruction**;** *LUTS*: lower urinary tract symptoms

# Supplementary Table 7. Extent of mono- and combination drug therapy use in the female LUTS (including OAB) sub-cohort

| **Monotherapy** | **N** | **% of monotherapy patients** | **% of all patients** | **Presence of a LUTS diagnosis Read code**  **n (%)** |
| --- | --- | --- | --- | --- |
| All monotherapy | 26338 | 100.0 | 90.5 | 3475 (13.2) |
| Solifenacin | 10083 | 38.3 | 34.7 | 1303 (12.9) |
| Oxybutynin | 7852 | 29.8 | 27.0 | 963 (12.3) |
| Tolterodine | 4020 | 15.3 | 13.8 | 511 (12.7) |
| Mirabegron | 2075 | 7.9 | 7.1 | 342 (16.5) |
| Fesoterodine | 1173 | 4.5 | 4.0 | 191 (16.3) |
| Trospium | 754 | 2.9 | 2.6 | 109 (14.5) |
| Darifenacin | 209 | 0.8 | 0.7 | 35 (16.7) |
| Flavoxate | 96 | 0.4 | 0.3 | 15 (15.6) |
| Propiverine | 76 | 0.3 | 0.3 | 6 (7.9) |
| **Combination drug therapy** | **N** | **% of combination drug therapy patients** | **% of all patients** | **Presence of a LUTS diagnosis Read code**  **n (%)** |
| All combination drug therapy | 2756 | 100.0 | 9.5 | 390 (14.2) |
| Solifenacin + tolterodine | 507 | 18.4 | 1.7 | 55 (10.8) |
| Oxybutynin + solifenacin | 458 | 16.6 | 1.6 | 66 (14.4) |
| Mirabegron + solifenacin | 417 | 15.1 | 1.4 | 72 (17.3) |
| Fesoterodine + solifenacin | 174 | 6.3 | 0.6 | 30 (17.2) |
| Oxybutynin + tolterodine | 174 | 6.3 | 0.6 | 23 (13.2) |
| Solifenacin + trospium | 127 | 4.6 | 0.4 | 26 (20.5) |
| Fesoterodine + mirabegron | 114 | 4.1 | 0.4 | 13 (11.4) |
| Mirabegron + tolterodine | 89 | 3.2 | 0.3 | 11 (12.4) |
| Mirabegron + oxybutynin | 83 | 3.0 | 0.3 | 12 (14.5) |
| Fesoterodine + tolterodine | 63 | 2.3 | 0.2 | 9 (14.3) |
| Duloxetine + solifenacin | 62 | 2.2 | 0.2 | 4 (6.5) |
| Tolterodine + trospium | 57 | 2.1 | 0.2 | 10 (17.5) |
| Darifenacin + solifenacin | 56 | 2.0 | 0.2 | 3 (5.4) |
| Mirabegron + trospium | 48 | 1.7 | 0.2 | 10 (20.8) |
| Oxybutynin + trospium | 47 | 1.7 | 0.2 | 10 (21.3) |
| Fesoterodine + oxybutynin | 39 | 1.4 | 0.1 | 4 (10.3) |
| Fesoterodine + trospium | 26 | 0.9 | 0.1 | 3 (11.5) |
| Duloxetine + tolterodine | 23 | 0.8 | 0.1 | 1 (4.3) |
| Propiverine + solifenacin | 20 | 0.7 | 0.1 | 1 (5.0) |
| Duloxetine + oxybutynin | 18 | 0.7 | 0.1 | 3 (16.7) |
| Other combinations | 154 | 5.6 | 0.5 | 24 (15.6) |

*LUTS*: lower urinary tract symptoms; *OAB*: overactive bladder

# Supplementary Table 8. Persistence in post-index period in the male BPO sub-cohort

|  | Index drug | N | Persistence days  Median (Q1 – Q3) |
| --- | --- | --- | --- |
| Monotherapy | Tamsulosin | 21158 | 329 (58–365^†^) |
|  | Doxazosin | 5456 | > 365* (163–365^†^) |
|  | Finasteride | 2131 | > 365* (84–365^†^) |
|  | Alfuzosin | 665 | 126 (34–365^†^) |
|  | Dutasteride | 146 | 305 (56–365^†^) |
|  | Prazosin | 101 | 201 (60–365^†^) |
|  | Terazosin | 44 | 150 (32–365^†^) |
|  | Indoramin | 38 | 192 (30–365^†^) |
| Combination drug therapy | Finasteride + tamsulosin | 3262 | > 365* (112–365^†^) |
|  | Dutasteride + tamsulosin | 1189 | > 365* (113–365^†^) |
|  | Doxazosin + tamsulosin | 614 | 109 (51–365^†^) |
|  | Doxazosin + finasteride | 289 | 339 (105–365^†^) |
|  | Alfuzosin + finasteride | 233 | 335 (66–365^†^) |
|  | Alfuzosin + tamsulosin | 208 | 56 (36.5–94.5) |
|  | Doxazosin + finasteride + tamsulosin | 167 | 107 (55–365^†^) |
|  | Dutasteride + finasteride + tamsulosin | 125 | 49 (30–69) |
|  | Doxazosin + dutasteride + tamsulosin | 55 | 223 (58–365^†^) |
|  | Dutasteride + finasteride | 46 | 48 (29–61) |
|  | Alfuzosin + dutasteride + tamsulosin | 44 | 65 (38.5–98) |
|  | Alfuzosin + finasteride + tamsulosin | 41 | 55 (30–88) |
|  | Tamsulosin + terazosin | 29 | 49 (31–82) |
|  | Alfuzosin + dutasteride | 25 | 249 (66–365^†^) |
|  | Finasteride + terazosin | 25 | 334 (59–365^†^) |
|  | Prazosin + tamsulosin | 23 | 87 (43–136) |
|  | Alfuzosin + doxazosin | 20 | 86 (34–213) |
|  | Indoramin + tamsulosin | 19 | 61 (45–103) |
|  | Doxazosin + dutasteride | 18 | > 365* (87–365^†^) |
|  | Other combinations | 136 | 66 (42.5–109) |

*BPO*: benign prostatic obstruction; *Q1*: lower quartile; *Q3*: upper quartile

^†^Q3 not reached by 365 days, *Median not reached by 365 days

# Supplementary Table 9. Persistence at 1 month, 6 months and 1 year in the male BPO population

|  | **N** | **1 month**  **% [95% CI]** | **6 months**  **% [95% CI]** | **1 year**  **% [95% CI]** | **Median (months)**  **[95% CI]** |
| --- | --- | --- | --- | --- | --- |
| **All**  Number of patients still at risk* | 36307 | 84.6 | 60.0 | 50.8 | 12.6 |
|  |  | [84.2, 85.0] | [59.5, 60.5] | [50.2, 51.3] | [12.2, 13.1] |
|  |  | 30721 | 21783 | 18357 |  |
| **Monotherapy**  Number of patients still at risk* | 29739 | 83.0 | 60.8 | 52.0 | 13.8 |
|  |  | [82.6, 83.4] | [60.3, 61.4] | [51.4, 52.5] | [13.2, 14.4] |
|  |  | 24689 | 18095 | 15390 |  |
| **Combination drug therapy**  Number of patients still at risk* | 6568 | 91.8 | 56.2 | 45.3 | 8.8 |
|  |  | [91.2, 92.5] | [54.9, 57.3] | [44.1, 46.5] | [8.2, 9.4] |
|  |  | 6032 | 3688 | 2967 |  |
| **Monotherapy** | | | | | |
| **Alfuzosin**    Number of patients still at risk* | 665 | 75.9 | 44.1 | 34.9 | 4.1 |
|  |  | [72.5, 79.0] | [40.3, 47.8] | [31.3, 38.5] | [3.1, 5.1] |
|  |  | 505 | 293 | 232 |  |
| **Doxazosin**    Number of patients still at risk* | 5456 | 89.0 | 74.1 | 67.8 | 38.5 |
|  |  | [88.1, 89.8] | [73.0, 75.3] | [66.6, 69.1] | [36.3, 42.7] |
|  |  | 4854 | 4045 | 3682 |  |
| **Dutasteride**    Number of patients still at risk* | 146 | 82.9 | 59.6 | 46.6 | 10 |
|  |  | [75.7, 88.1] | [51.2, 67.0] | [38.3, 54.4] | [6.1, 13.8] |
|  |  | 121 | 87 | 68 |  |
| **Finasteride**    Number of patients still at risk* | 2131 | 87.8 | 64.0 | 53.3 | 14.8 |
|  |  | [86.3, 89.1] | [61.9, 66.0] | [51.1, 55.3] | [12.9, 17.6] |
|  |  | 1871 | 1364 | 1126 |  |
| **Indoramin**    Number of patients still at risk* | 38 | 73.7 | 52.6 | 42.1 | 6.3 |
|  |  | [56.6, 84.9] | [35.8, 67.0] | [26.4, 57.0] | [2.8, 17.2] |
|  |  | 28 | 20 | 16 |  |
| **Prazosin**    Number of patients still at risk* | 101 | 84.2 | 51.5 | 45.5 | 6.6 |
|  |  | [75.5, 90.0] | [41.4, 60.7] | [35.6, 54.9] | [3.8, 16.6] |
|  |  | 85 | 52 | 46 |  |
| **Tamsulosin**    Number of patients still at risk* | 21158 | 81.3 | 57.7 | 48.4 | 10.8 |
|  |  | [80.7, 81.8] | [57.0, 58.4] | [47.7, 49.1] | [10.3, 11.3] |
|  |  | 17192 | 12212 | 10204 |  |
| **Terazosin**    Number of patients still at risk* | 44 | 75.0 | 50.0 | 36.4 | 4.9 |
|  |  | [59.4, 85.3] | [34.6, 63.6] | [22.6, 50.3] | [1.8, 12.8] |
|  |  | 33 | 22 | 16 |  |
| **Combination drug therapy** | | | | | |
| **Alfuzosin + doxazosin**    Number of patients still at risk* | 20 | 85.0 | 25.0 | NO | 2.8 |
|  |  | [60.4, 94.9] | [9.1, 44.9] | — | [1.1, 4.3] |
|  |  | 17 | 5 | 4 |  |
| **Alfuzosin + dutasteride**    Number of patients still at risk* | 25 | 96.0 | 52.0 | 44.0 | 8.2 |
|  |  | [74.8, 99.4] | [31.2, 69.2] | [24.5, 61.9] | [2.3, 18.8] |
|  |  | 24 | 13 | 11 |  |
| **Alfuzosin + dutasteride  + tamsulosin**    Number of patients still at risk* | 44 | 84.1 | NO | NO | 2.1 |
|  |  | [69.5, 92.1] | — | — | [1.6, 2.7] |
|  |  | 37 | 3 | 2 |  |
| **Alfuzosin + finasteride**    Number of patients still at risk* | 233 | 92.3 | 59.2 | 48.9 | 11 |
|  |  | [88.0, 95.1] | [52.6, 65.2] | [42.4, 55.2] | [6.8, 16.0] |
|  |  | 215 | 138 | 114 |  |
| **Alfuzosin + finasteride  + tamsulosin**  Number of patients still at risk* | 41 | 73.2 | NO | NO | 1.8 |
|  |  | [56.8, 84.1] | — | — | [1.1, 2.5] |
|  |  | 30 | 1 | 1 |  |
| **Alfuzosin + tamsulosin**    Number of patients still at risk* | 208 | 83.7 | NO | NO | 1.8 |
|  |  | [77.9, 88.0] | — | — | [1.7, 2.0] |
|  |  | 174 | 8 | 4 |  |
| **Doxazosin + dutasteride**    Number of patients still at risk* | 18 | 100.0 | 66.7 | 55.6 | 12.9 |
|  |  | — | [40.4, 83.4] | [30.5, 74.8] | [2.9, 18.2] |
|  |  | 18 | 12 | 10 |  |
| **Doxazosin + dutasteride  + tamsulosin**    Number of patients still at risk* | 55 | 92.7 | 54.5 | 40.0 | 7.3 |
|  |  | [81.8, 97.2] | [40.6, 66.6] | [27.1, 52.5] | [2.6, 13.0] |
|  |  | 51 | 30 | 22 |  |
| **Doxazosin + finasteride**    Number of patients still at risk* | 289 | 95.5 | 63.3 | 48.8 | 11.1 |
|  |  | [92.4, 97.4] | [57.5, 68.6] | [42.9, 54.4] | [8.0, 13.8] |
|  |  | 276 | 183 | 140 |  |
| **Doxazosin + finasteride  + tamsulosin**  Number of patients still at risk* | 167 | 95.2 | 37.7 | 31.1 | 3.5 |
|  |  | [90.6; 97.6] | [30.4, 45.0] | [24.3, 38.2] | [2.9, 4.4] |
|  |  | 159 | 63 | 52 |  |
| **Doxazosin + tamsulosin**    Number of patients still at risk* | 614 | 87.9 | 38.1 | 28.7 | 3.6 |
|  |  | [85.1, 90.3] | [34.3, 41.9] | [25.1, 32.3] | [2.9, 3.9] |
|  |  | 540 | 234 | 176 |  |
| **Dutasteride + finasteride**    Number of patients still at risk* | 46 | 71.7 | NO | NO | 1.6 |
|  |  | [56.4, 82.5] | — | — | [1.0, 1.8] |
|  |  | 33 | 0 | 0 |  |
| **Dutasteride + finasteride  + tamsulosin**    Number of patients still at risk* | 125 | 74.4 | NO | NO | 1.6 |
|  |  | [65.8, 81.2] | — | — | [1.4, 1.8] |
|  |  | 93 | 4 | 1 |  |
| **Dutasteride + tamsulosin**    Number of patients still at risk* | 1189 | 89.8 | 68.0 | 56.3 | 18 |
|  |  | [88.0, 91.4] | [65.3, 70.6] | [53.5, 59.1] | [15.4, 20.8] |
|  |  | 1068 | 809 | 668 |  |
| **Finasteride + indoramin**    Number of patients still at risk* | 15 | 86.7 | 60.0 | 53.3 | 13.8 |
|  |  | [56.4, 96.5] | [31.8, 79.7] | [26.3, 74.4] | [2.3, — ] |
|  |  | 13 | 9 | 8 |  |
| **Finasteride + tamsulosin**    Number of patients still at risk* | 3262 | 95.1 | 65.7 | 53.1 | 13.6 |
|  |  | [94.3, 95.8] | [64.0, 67.3] | [51.4, 54.8] | [12.7, 14.9] |
|  |  | 3101 | 2143 | 1728 |  |
| **Finasteride + terazosin**    Number of patients still at risk* | 25 | 92.0 | 56.0 | 48.0 | 11 |
|  |  | [71.6, 97.9] | [34.8, 72.7] | [27.8, 65.6] | [2.8, 23.2] |
|  |  | 23 | 14 | 12 |  |
| **Indoramin + tamsulosin**    Number of patients still at risk* | 19 | 94.7 | NO | NO | 2 |
|  |  | [68.1, 99.2] | — | — | [1.5, 2.9] |
|  |  | 18 | 1 | 0 |  |
| **Prazosin + tamsulosin**    Number of patients still at risk* | 23 | 78.3 | NO | NO | 2.9 |
|  |  | [55.4, 90.3] | — | — | [1.8, 3.6] |
|  |  | 18 | 3 | 3 |  |
| **Tamsulosin + terazosin**    Number of patients still at risk* | 29 | 79.3 | NO | NO | 1.6 |
|  |  | [59.6, 90.1] | — | — | [1.1, 2.2] |
|  |  | 23 | 1 | 1 |  |
| **Other combinations**    Number of patients still at risk* | 121 | 83.5 | NO | NO | 2 |
|  |  | [75.6, 89.0] | — | — | [1.7, 2.3] |
|  |  | 101 | 14 | 10 |  |

*BPO*: benign prostatic obstruction; *CI*: confidence interval, *NO*: not observable

*Number of patients still observable at a given time and for whom no events occurred.

Not Observable indicates that the number of patients still at risk was below the 20% of the initial sample threshold required to calculate persistence, or the median was not reached.

# Supplementary Table 10. Persistence in post-index period in the female LUTS (including OAB) sub-cohort

|  | **Index drug** | **N** | **Persistence days**  **Median (Q1 - Q3)** |
| --- | --- | --- | --- |
| **Monotherapy** | Solifenacin | 10083 | 127 (30–365^†^) |
|  | Oxybutynin | 7852 | 57 (28–365^†^) |
|  | Tolterodine | 4020 | 82 (28–365^†^) |
|  | Mirabegron | 2075 | 244 (42–365^†^) |
|  | Fesoterodine | 1173 | 105 (28–365^†^) |
|  | Trospium | 754 | 89 (30–365^†^) |
|  | Darifenacin | 209 | 167 (29–365^†^) |
|  | Flavoxate | 96 | 30 (30–85) |
|  | Propiverine | 76 | 52 (28–365^†^) |
| **Combination drug therapy** | Solifenacin + tolterodine | 507 | 48 (30–69) |
|  | Oxybutynin + solifenacin | 458 | 51 (34–72) |
|  | Mirabegron + solifenacin | 417 | 57 (36–105) |
|  | Fesoterodine + solifenacin | 174 | 49 (31–83) |
|  | Oxybutynin + tolterodine | 174 | 45 (29–67) |
|  | Solifenacin + trospium | 127 | 50 (33–68) |
|  | Fesoterodine + mirabegron | 114 | 70 (40–116) |
|  | Mirabegron + tolterodine | 89 | 52 (36–86) |
|  | Mirabegron + oxybutynin | 83 | 50 (37–65) |
|  | Fesoterodine + tolterodine | 63 | 48 (31–57) |
|  | Duloxetine + solifenacin | 62 | 61 (31–188) |
|  | Tolterodine + trospium | 57 | 46 (30–69) |
|  | Darifenacin + solifenacin | 56 | 34 (28–46) |
|  | Mirabegron + trospium | 48 | 56 (41–98.5) |
|  | Oxybutynin + trospium | 47 | 42 (28–60) |
|  | Fesoterodine + oxybutynin | 39 | 61 (47–100) |
|  | Fesoterodine + trospium | 26 | 48 (40–76) |
|  | Duloxetine + tolterodine | 23 | 69 (28–116) |
|  | Propiverine + solifenacin | 20 | 39 (27.5–48.5) |
|  | Other combinations | 172 | 51 (34–80) |

*LUTS*: lower urinary tract symptoms; *OAB*: overactive bladder; *Q1*: lower quartile; *Q3*: upper quartile

^†^Q3 not reached by 365 days

# Supplementary Table 11. Persistence at 1 month, 6 months and 1 year in female LUTS (including OAB) population

|  | **N** | | **1 month**  **% [95% CI]** | **6 months**  **% [95% CI]** | **1 year**  **% [95% CI]** | **Median (months)**  **[95% CI]** |
| --- | --- | --- | --- | --- | --- | --- |
| **All**  Number of patients still at risk* | 29094 | | 67.1 | 38.4 | 29.6 | 2.6 |
|  |  | | [66.5, 67.6] | [37.8, 38.9] | [29.1, 30.1] | [2.5, 2.7] |
|  |  | | 19519 | 11168 | 8573 |  |
| **Monotherapy**  Number of patients still at risk* | 26338 | | 66.0 | 41.7 | 32.2 | 3.1 |
|  |  | | [65.5, 66.6] | [41.1, 42.3] | [31.7, 32.8] | [3.0, 3.3] |
|  |  | | 17394 | 10973 | 8445 |  |
| **Combination drug therapy**  Number of patients still at risk* | 2756 | | 77.1 | NO | NO | 1.6 |
|  |  | | [75.5, 78.6] | — | — | [1.6, 1.7] |
|  |  | | 2125 | 195 | 128 |  |
| **Monotherapy** |  |  |  |  |  |  |
| **Darifenacin**    Number of patients still at risk* | 209 | | 73.2 | 49.3 | 38.3 | 5.5 |
|  |  | | [66.7, 78.7] | [42.3, 55.8] | [31.7, 44.8] | [3.9, 8.5] |
|  |  | | 153 | 103 | 79 |  |
|  |  | |  |  |  |  |
| **Fesoterodine**    Number of patients still at risk* | 1173 | | 67.7 | 42.7 | 31.3 | 3.4 |
|  |  | | [64.9, 70.3] | [39.9, 45.5] | [28.7, 34.0] | [2.8, 4.1] |
|  |  | | 794 | 501 | 367 |  |
|  |  | |  |  |  |  |
| **Flavoxate**    Number of patients still at risk* | 96 | | 41.7 | 19.8 | NO | 1 |
|  |  | | [31.8, 51.3] | [12.5, 28.3] | — | [1.0, 1.1] |
|  |  | | 40 | 19 | 12 |  |
|  |  | |  |  |  |  |
| **Mirabegron**    Number of patients still at risk* | 2075 | | 76.5 | 54.1 | 43.5 | 8 |
|  |  | | [74.6, 78.3] | [51.9, 56.2] | [41.3, 45.6] | [6.9, 9.3] |
|  |  | | 1588 | 1122 | 899 |  |
|  |  | |  |  |  |  |
| **Oxybutynin**    Number of patients still at risk* | 7852 | | 60.4 | 35.2 | 27.3 | 1.9 |
|  |  | | [59.3, 61.5] | [34.1, 36.2] | [26.3, 28.3] | [1.8, 2.0] |
|  |  | | 4743 | 2763 | 2140 |  |
|  |  | |  |  |  |  |
| **Propiverine**    Number of patients still at risk* | 76 | | 64.5 | 35.5 | 30.3 | 1.7 |
|  |  | | [52.6, 74.1] | [25.0, 46.2] | [20.4, 40.7] | [1.2, 3.2] |
|  |  | | 49 | 27 | 23 |  |
|  |  | |  |  |  |  |
| **Solifenacin**    Number of patients still at risk* | 10083 | | 68.9 | 45.0 | 34.8 | 4.2 |
|  |  | | [68.0, 69.8] | [44.0, 46.0] | [33.8, 35.7] | [3.9, 4.4] |
|  |  | | 6948 | 4539 | 3483 |  |
|  |  | |  |  |  |  |
| **Tolterodine**    Number of patients still at risk* | 4020 | | 64.0 | 39.7 | 30.4 | 2.7 |
|  |  | | [62.5, 65.5] | [38.2, 41.2] | [29.0, 31.8] | [2.4, 3.0] |
|  |  | | 2574 | 1595 | 1212 |  |
|  |  | |  |  |  |  |
| **Trospium**    Number of patients still at risk* | 754 | | 67.0 | 40.3 | 30.8 | 2.9 |
|  |  | | [63.5, 70.2] | [36.8, 43.8] | [27.5, 34.1] | [2.4, 3.8] |
|  |  | | 505 | 304 | 230 |  |
|  |  | |  |  |  |  |
| **Combination drug therapy** | |  |  |  |  |  |
| **Darifenacin + solifenacin**    Number of patients still at risk* | 56 | | 60.7 | NO | NO | 1.1 |
|  |  | | [46.7, 72.1] | — | — | [1.0, 1.3] |
|  |  | | 34 | 1 | 0 |  |
|  |  | |  |  |  |  |
| **Duloxetine + oxybutynin**    Number of patients still at risk* | 18 | | 88.9 | 27.8 | NO | 2.0 |
|  |  | | [62.4, 97.1] | [10.1, 48.9] |  | [1.4, 4.1] |
|  |  | | 16 | 5 | 2 |  |
|  |  | |  |  |  |  |
| **Duloxetine + solifenacin**    Number of patients still at risk* | 62 | | 77.4 | 27.4 | NO | 2.0 |
|  |  | | [64.9, 86.0] | [17.0, 38.8] | — | [1.6, 2.9] |
|  |  | | 48 | 17 | 10 |  |
|  |  | |  |  |  |  |
| **Duloxetine + tolterodine**    Number of patients still at risk* | 23 | | 73.9 | NO | NO | 2.3 |
|  |  | | [50.9, 87.3] | — | — | [1.4, 3.5] |
|  |  | | 17 | 4 | 1 |  |
|  |  | |  |  |  |  |
| **Fesoterodine + mirabegron**    Number of patients still at risk* | 114 | | 80.7 | 17.5 | NO | 2.3 |
|  |  | | [72.2, 86.8] | [11.2, 25.0] | — | [1.8, 2.8] |
|  |  | | 92 | 20 | 17 |  |
|  |  | |  |  |  |  |
| **Fesoterodine + oxybutynin**    Number of patients still at risk* | 39 | | 87.2 | NO | NO | 2 |
|  |  | | [71.9, 94.5] | — | — | [1.7, 2.9] |
|  |  | | 34 | 1 | 0 |  |
|  |  | |  |  |  |  |
| **Fesoterodine + solifenacin**    Number of patients still at risk* | 174 | | 75.3 | NO | NO | 1.6 |
|  |  | | [68.2, 81.0] | — | — | [1.4, 1.8] |
|  |  | | 131 | 7 | 5 |  |
|  |  | |  |  |  |  |
| **Fesoterodine + tolterodine**    Number of patients still at risk* | 63 | | 76.2 | NO | NO | 1.6 |
|  |  | | [63.6, 84.9] | — | — | [1.2, 1.7] |
|  |  | | 48 | 1 | 0 |  |
|  |  | |  |  |  |  |
| **Fesoterodine + trospium**    Number of patients still at risk* | 26 | | 80.8 | NO | NO | 1.6 |
|  |  | | [59.8, 91.5] | — | — | [1.3, 2.0] |
|  |  | | 21 | 1 | 0 |  |
|  |  | |  |  |  |  |
| **Mirabegron + oxybutynin**    Number of patients still at risk* | 83 | | 84.3 | NO | NO | 1.6 |
|  |  | | [74.6, 90.6] | — | — | [1.5, 1.8] |
|  |  | | 70 | 9 | 9 |  |
|  |  | |  |  |  |  |
| **Mirabegron + solifenacin**    Number of patients still at risk* | 417 | | 80.3 | 17.0 | NO | 1.9 |
|  |  | | [76.2, 83.8] | [13.6, 20.8] | — | [1.8, 2.1] |
|  |  | | 335 | 71 | 49 |  |
|  |  | |  |  |  |  |
| **Mirabegron + tolterodine**    Number of patients still at risk* | 89 | | 79.8 | NO | NO | 1.7 |
|  |  | | [69.8, 86.7] | — | — | [1.5, 1.9] |
|  |  | | 71 | 6 | 4 |  |
|  |  | |  |  |  |  |
| **Mirabegron + trospium**    Number of patients still at risk* | 48 | | 87.5 | NO | NO | 1.8 |
|  |  | | [74.3, 94.2] | — | — | [1.5, 2.6] |
|  |  | | 42 | 7 | 5 |  |
|  |  | |  |  |  |  |
| **Oxybutynin + solifenacin**    Number of patients still at risk* | 458 | | 79.9 | NO | NO | 1.7 |
|  |  | | [75.9, 83.3] | — | — | [1.6, 1.8] |
|  |  | | 366 | 14 | 8 |  |
| **Oxybutynin + tolterodine**   Number of patients still at risk* | 174 | | 72.4 | NO | NO | 1.5 |
|  |  | | [65.1, 78.4] | — | — | [1.3, 1.6] |
|  |  | | 126 | 5 | 2 |  |
|  |  | |  |  |  |  |
| **Oxybutynin + trospium**  Number of patients still at risk* | 47 | | 72.3 | NO | NO | 1.4 |
|  |  | | [57.2, 82.9] | — | — | [1.1, 1.7] |
|  |  | | 34 | 0 | 0 |  |
|  |  | |  |  |  |  |
| **Other combinations**   Number of patients still at risk* | 154 | | 75.3 | NO | NO | 1.6 |
|  |  | | [67.7, 81.4] | — | — | [1.4, 1.7] |
|  |  | | 116 | 16 | 11 |  |
|  |  | |  |  |  |  |
| **Propiverine + solifenacin**  Number of patients still at risk* | 20 | | 60.0 | NO | NO | 1.3 |
|  |  | | [35.7, 77.6] | — | — | [0.9, 1.6] |
|  |  | | 12 | 0 | 0 |  |
|  |  | |  |  |  |  |
| **Solifenacin + tolterodine**    Number of patients still at risk* | 507 | | 73.4 | NO | NO | 1.6 |
|  |  | | [69.3, 77.0] | — | — | [1.5, 1.6] |
|  |  | | 372 | 4 | 2 |  |
|  |  | |  |  |  |  |
| **Solifenacin + trospium**  Number of patients still at risk* | 127 | | 77.2 | NO | NO | 1.6 |
|  |  | | [68.8, 83.5] | — | — | [1.5, 1.9] |
|  |  | | 98 | 5 | 3 |  |
|  |  | |  |  |  |  |
| **Tolterodine + trospium**    Number of patients still at risk* | 57 | | 73.7 | NO | NO | 1.5 |
|  |  | | [60.2, 83.2] | — | — | [1.3, 1.7] |
|  |  | | 42 | 1 | 0 |  |

*CI*: confidence interval; *LUTS*: lower urinary tract symptoms; *NO*: not observable; *OAB*: overactive bladder

*Number of patients still observable at a given time and for whom no events occurred.

Not Observable indicates that the number of patients still at risk was below the 20% of the initial sample threshold required to calculate persistence, or the median was not reached.

# Supplementary Table 12. Extent of mono- and combination drug therapy use in the male BPO sub-cohort - Sensitivity analyses

|  | **Main analysis** | **SA1** | **SA2** | **SA3** | **SA4** | **SA5** | **SA6** | **SA7** |
| --- | --- | --- | --- | --- | --- | --- | --- | --- |
| **Monotherapy** | **Proportions of all monotherapy, %** | | | | | | | |
| Tamsulosin | 71.1 | 71.4 | 66.5 | 71.1 | 71.1 | 71.2 | 91.9 | 79.3 |
| Doxazosin | 18.3 | 18.6 | 16.9 | 18.3 | 18.3 | 18.4 | 1.6 | 8.9 |
| Finasteride | 7.2 | 6.9 | 12.4 | 7.2 | 7.2 | 7.1 | 2.7 | 8.1 |
| Alfuzosin | 2.2 | 2.2 | 2.6 | 2.2 | 2.3 | 2.2 | 2.9 | 2.6 |
| Dutasteride | 0.5 | 0.5 | 0.9 | 0.5 | 0.5 | 0.5 | 0.6 | 0.5 |
| Prazosin | 0.3 | 0.3 | 0.3 | 0.3 | 0.3 | 0.3 | 0.1 | 0.4 |
| Terazosin | 0.1 | 0.1 | 0.2 | 0.1 | 0.1 | 0.1 | 0.1 | 0.2 |
| Indoramin | 0.1 | 0.1 | 0.2 | 0.1 | 0.1 | 0.1 | 0.1 | 0.1 |
| **Combination drug therapy** | **Proportions of all combination drug therapy, %** | | | | | | | |
| Finasteride + tamsulosin | 49.7 | 48.8 | 50.8 | 56.4 | 49.9 | 49.6 | 44.1 | 53.1 |
| Dutasteride + tamsulosin | 18.1 | 17.0 | 37.1 | 20.1 | 18.4 | 18.0 | 26.0 | 19.0 |
| Doxazosin + tamsulosin | 9.3 | 9.6 | 3.0 | 7.2 | 9.3 | 9.3 | 10.2 | 5.8 |
| Doxazosin + finasteride | 4.4 | 4.3 | 2.9 | 4.9 | 4.4 | 4.4 | 1.9 | 3.5 |
| Alfuzosin + finasteride | 3.5 | 3.4 | 3.2 | 3.6 | 3.5 | 3.5 | 2.6 | 4.0 |
| Alfuzosin + tamsulosin | 3.2 | 3.5 | 0.4 | 1.3 | 3.0 | 3.2 | 4.5 | 3.4 |
| Doxazosin + finasteride + tamsulosin | 2.5 | 2.7 | 0.8 | 2.0 | 2.5 | 2.5 | 2.2 | 1.6 |
| Dutasteride + finasteride + tamsulosin | 1.9 | 2.3 | 0.2 | 0.5 | 1.7 | 2.0 | 1.6 | 2.3 |
| Doxazosin + dutasteride + tamsulosin | 0.8 | 0.9 | 0.2 | 0.7 | 0.8 | 0.8 | 0.9 | 0.5 |
| Dutasteride + finasteride | 0.7 | 1.1 | 0.1 | 0.1 | 0.6 | 0.7 | 0.3 | 0.8 |
| Alfuzosin + dutasteride + tamsulosin | 0.7 | 0.7 | 0.0 | 0.3 | 0.6 | 0.7 | 1.0 | 0.7 |
| Alfuzosin + finasteride + tamsulosin | 0.6 | 0.7 | 0.1 | 0.2 | 0.6 | 0.6 | 0.9 | 0.8 |
| Tamsulosin + terazosin | 0.4 | 0.5 | 0.0 | 0.2 | 0.4 | 0.4 | 0.5 | 0.5 |
| Alfuzosin + dutasteride | 0.4 | 0.4 | 0.3 | 0.3 | 0.4 | 0.4 | 0.5 | 0.6 |
| Finasteride + terazosin | 0.4 | 0.3 | 0.2 | 0.4 | 0.4 | 0.4 | 0.3 | 0.4 |
| Prazosin + tamsulosin | 0.4 | 0.3 | 0.0 | 0.2 | 0.4 | 0.3 | 0.5 | 0.3 |
| Alfuzosin + doxazosin | 0.3 | 0.4 | 0.1 | 0.2 | 0.3 | 0.3 | 0.3 | 0.2 |
| Indoramin + tamsulosin | 0.3 | 0.4 | 0.1 | 0.0 | 0.2 | 0.3 | 0.3 | 0.4 |
| Doxazosin + dutasteride | 0.3 | 0.3 | 0.1 | 0.3 | 0.3 | 0.3 | 0.2 | 0.0 |
| Finasteride + indoramin | 0.2 | 0.0 | 0.2 | 0.2 | 0.2 | 0.2 | 0.0 | 0.3 |
| Other combinations | 1.8 | 2.1 | 0.2 | 0.7 | 1.8 | 1.9 | 1.2 | 1.7 |

*BPO*: benign prostatic obstruction; *SA*: sensitivity analysis

# Supplementary Figure 1. TTD for monotherapies in male BPO sub-cohort (Kaplan-Meier estimates)


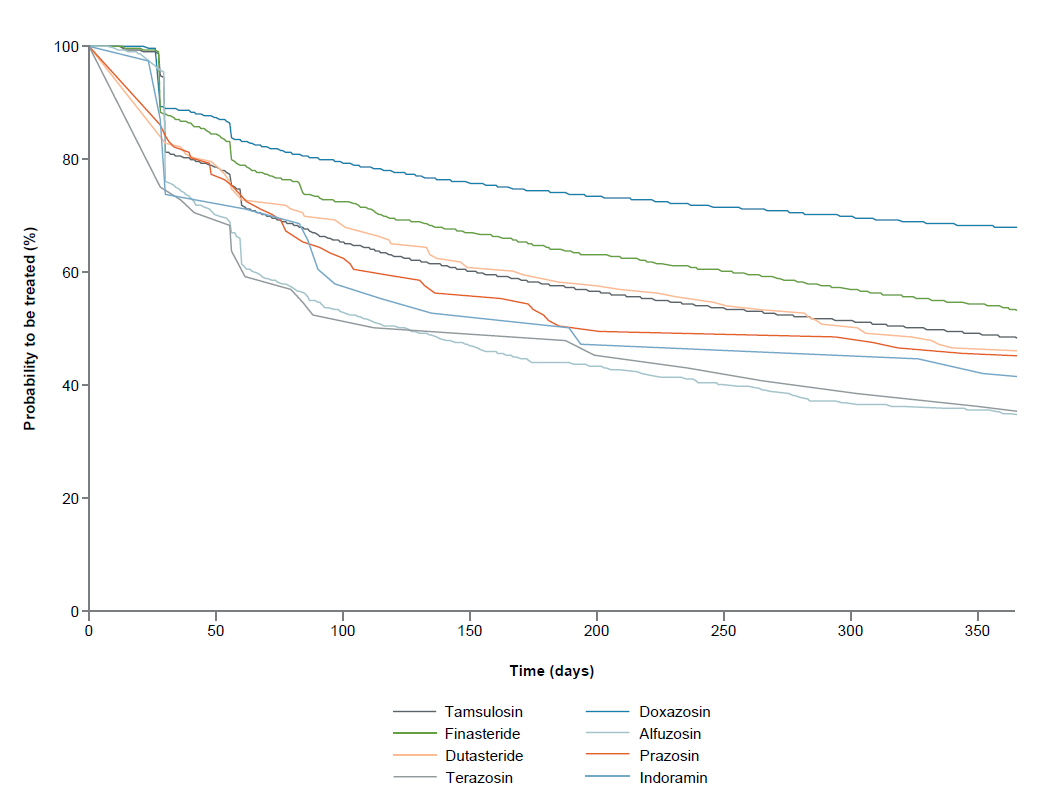


*BPO*: benign prostatic obstruction; *TTD*: time to discontinuation

# Supplementary Figure 2. TTD for combinations in male BPO sub-cohort (Kaplan-Meier estimates)^†^


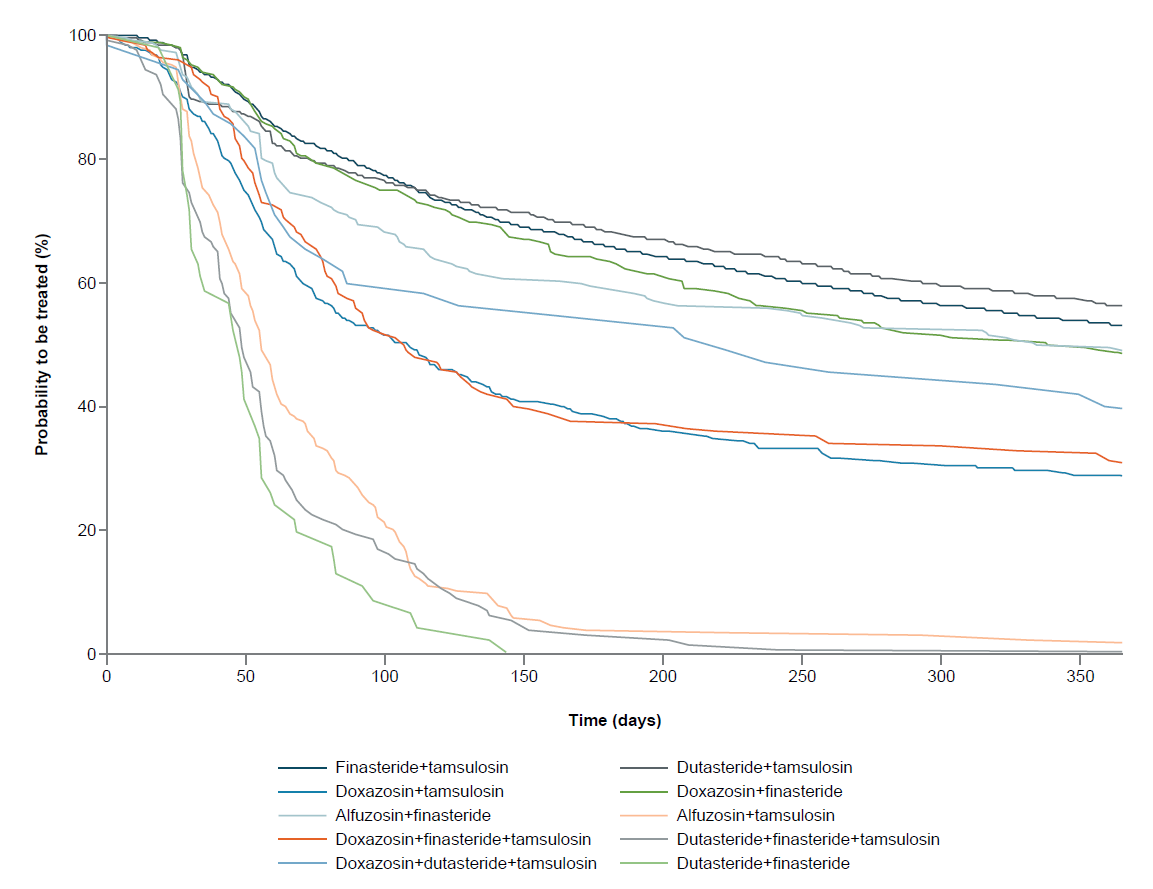


*BPO*: benign prostatic obstruction; *TTD*: time to discontinuation

^†^The 10 most frequent combination drug therapies have been plotted

# Supplementary Figure 3. TTD for duloxetine in female SUI sub-cohort (Kaplan-Meier estimates)


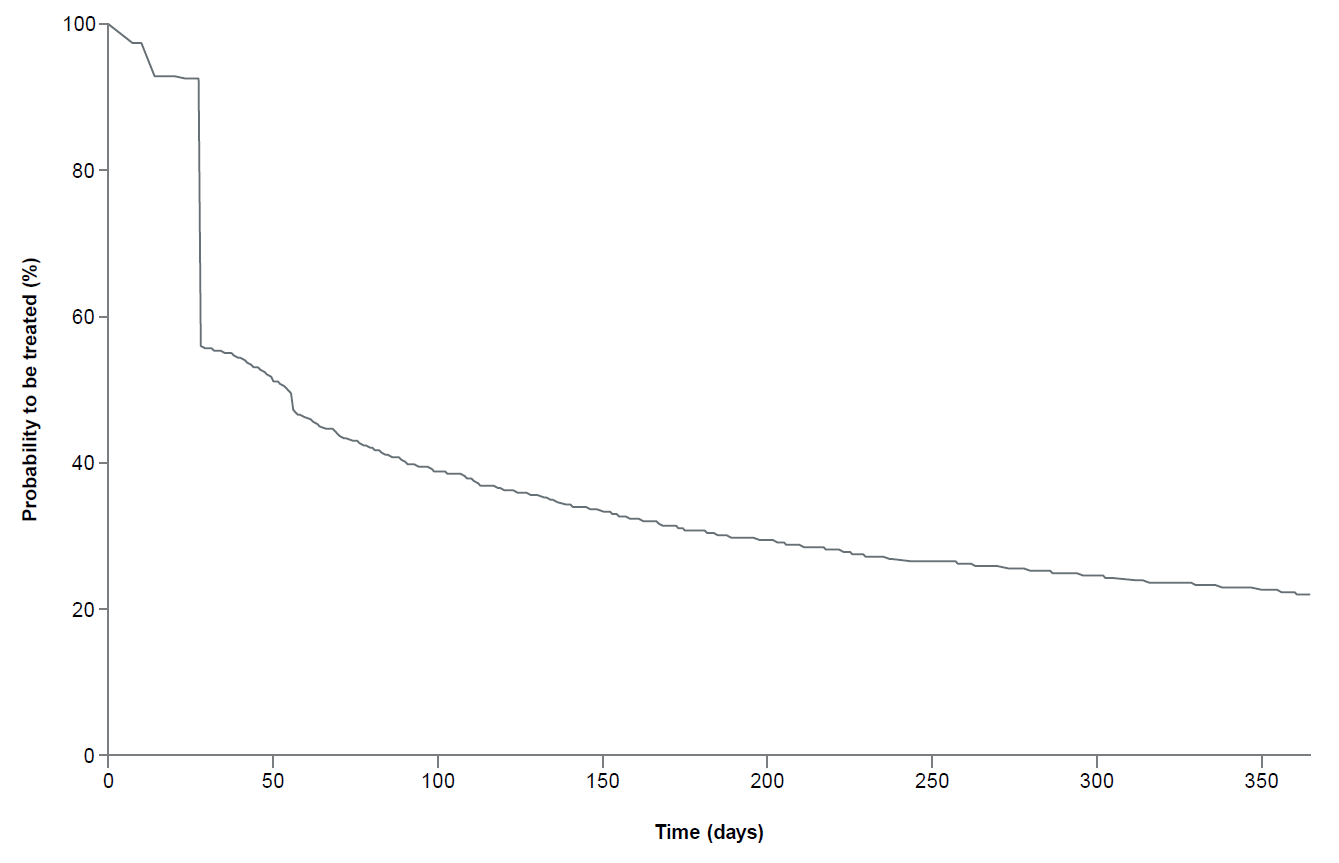


*SUI*: stress urinary incontinence; *TTD*: time to discontinuation

# Supplementary Figure 4. Kaplan-Meier curves for TTD sensitivity analysis: (A) monotherapy; (B) combination drug therapy (male BPO/LUTS population^†^)

**A**

**
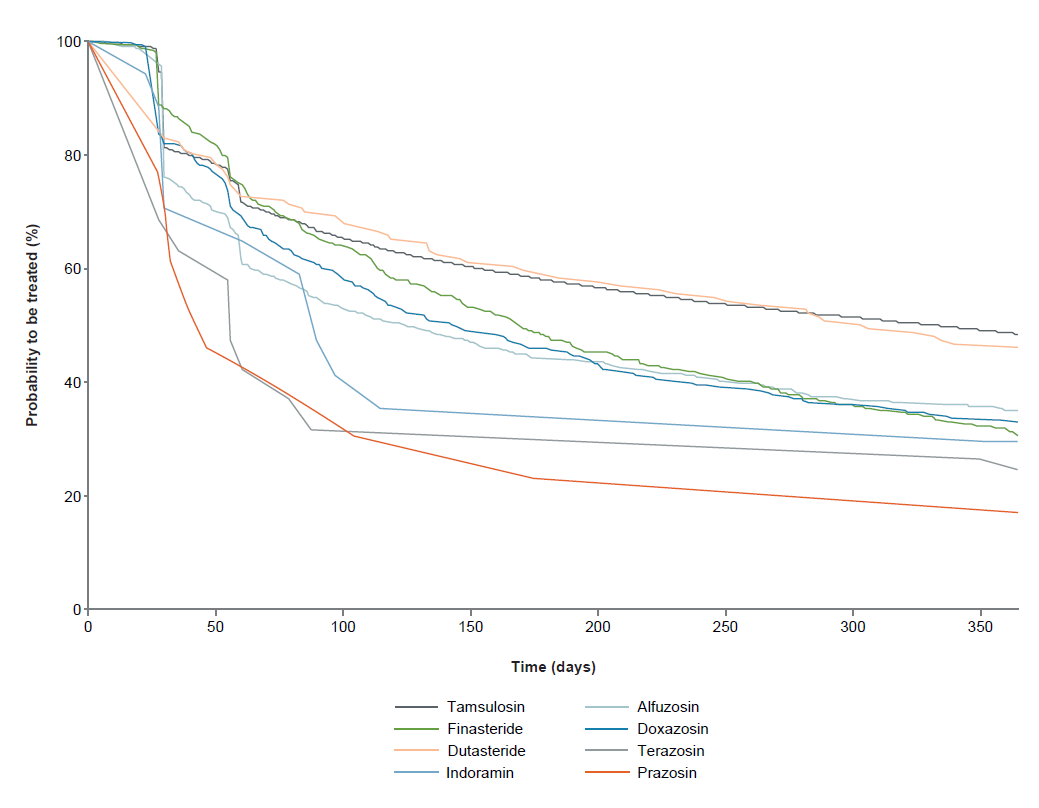
**

**B**

**
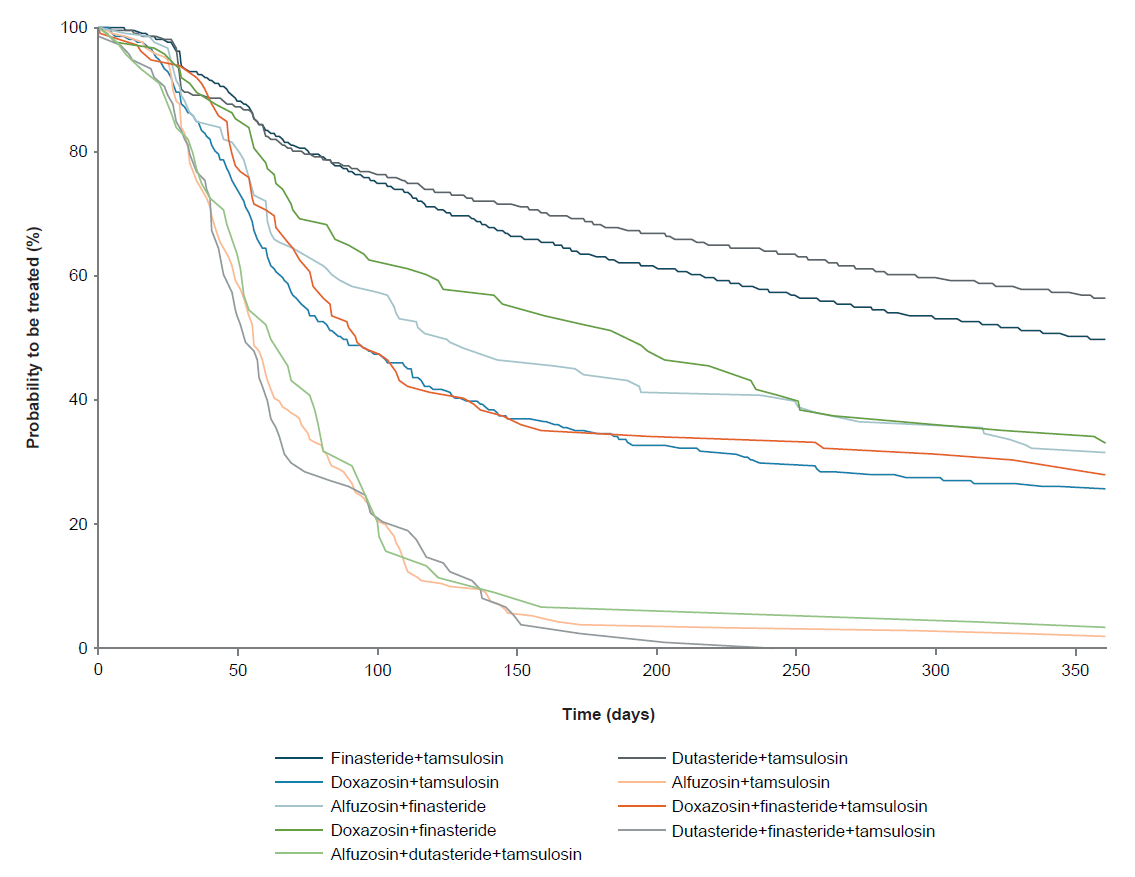
**

*BPO*: benign prostatic obstruction; *LUTS*: lower urinary tract symptoms; *TTD*: time to discontinuation

^†^patients with a confirmed LUTS diagnosis only

**References**

1. Morant SV, Reilly K, Bloomfield GA, Chapple C: **Diagnosis and treatment of lower urinary tract symptoms suggestive of overactive bladder and bladder outlet obstruction among men in general practice in the UK**. *Int J Clin Pract* 2008, **62**(5):688-694.
